# Discovery of 3-((3-amino-*1H*-indazol-4-yl)ethynyl)-*N*-(4-((4-ethylpiperazin-1-yl)methyl)-3-(trifluoromethyl)phenyl)benzamide (AKE-72), a potent Pan-BCR-ABL inhibitor including the T315I gatekeeper resistant mutant

**DOI:** 10.1080/14756366.2023.2228515

**Published:** 2023-07-20

**Authors:** Ashraf K. El-Damasy, Hyun Ji Kim, Jung Woo Park, Yunju Nam, Wooyoung Hur, Eun-Kyoung Bang, Gyochang Keum

**Affiliations:** a Brain Science Institute, Korea Institute of Science and Technology (KIST), Seoul, Republic of Korea; b Department of Medicinal Chemistry, Faculty of Pharmacy, Mansoura University, Mansoura, Egypt; c Supercomputing Application Center, Div. of National Supercomputing, Korea Institute of Science and Technology Information, Daejeon, Republic of Korea; d Medicinal Materials Research Center, Korea Institute of Science and Technology (KIST), Seoul, Republic of Korea; e Division of Bio-Medical Science & Technology, KIST School, Korea University of Science and Technology (UST), Seoul, Republic of Korea

**Keywords:** Chronic myeloid leukaemia, aminoindazole, diarylamides, ABL^T315I^, Imatinib resistance

## Abstract

BCR-ABL inhibition is an effective therapeutic approach for the treatment of chronic myeloid leukaemia (CML). Herein, we report the discovery of **AKE-72 (5)**, a diarylamide 3-aminoindazole, as a potent pan-BCR-ABL inhibitor, including the imatinib-resistant mutant T315I. A focussed array of compounds **4a**, **4b**, and **5** has been designed based on our previously reported indazole **I** to improve its BCR-ABL^T315I^ inhibitory activity. Replacing the morpholine moiety of **I** with the privileged tail (4-ethylpiperazin-1-yl)methyl afforded **5 (AKE-72)** with IC_50_ values of < 0.5 nM, and 9 nM against BCR-ABL^WT^ and BCR-ABL^T315I^, respectively. Moreover, **AKE-72** potently inhibited a panel of other clinically important mutants in single-digit nanomolar IC_50_ values. **AKE-72** elicited remarkable anti-leukemic activity against K-562 cell line (GI_50_ < 10 nM, TGI = 154 nM). In addition, **AKE-72** strongly inhibited the proliferation of Ba/F3 cells expressing native BCR-ABL or its T315I mutant. Overall, **AKE-72** may serve as a promising candidate for the treatment of CML, including those harbouring T315I mutation.

## GRAPHICAL ABSTRACT

**Figure:**
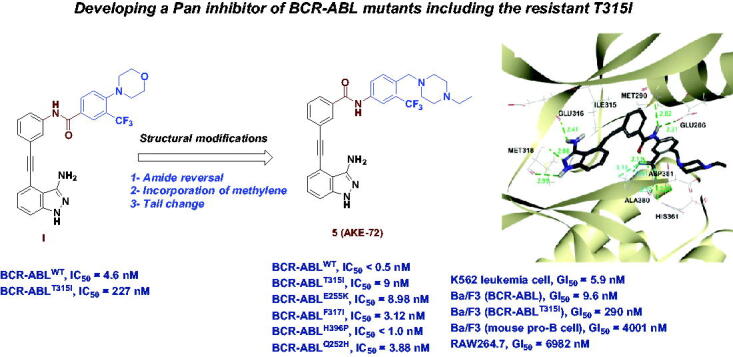

## Introduction

Chronic myeloid leukaemia (CML) is a myeloproliferative tumour of white blood cells in bone marrow and accounts for 15% of adult leukaemia^1^. Break-point cluster region-Abelson (BCR-ABL) is a fusion protein generated from the Philadelphia chromosome (Ph), which drives the pathogenesis of most of CML as well as a subset acute lymphoblastic leukaemia (Ph-positive ALL)^2^^,^^3^. Therefore, the design of kinase inhibitors targeting the BCR-ABL oncoprotein represents an effective strategy for the therapy of CML and/or ALL.

Imatinib, the first targeted therapy drug, is a first-line BCR-ABL inhibitor, which showed remarkable efficacy for most patients diagnosed with CML, with an overall 5-year survival rate of 89%^4^^,^^5^. Imatinib therapy for CML was estimated to have a long-term success rate of 83.3% after 10 years^6^. Nevertheless, the dose intolerance and acquired resistance to imatinib, through the emergence of point mutations, hampered its efficacy for CML patients in accelerated and blastic phases^5^^,^^7^. Nilotinib, bafetinib, dasatinib, and bosutinib have been developed as second-generation BCR-ABL inhibitors, with superior potency over imatinib, to treat adult CML patients with imatinib resistance^8–11^. However, these drugs are effective against most imatinib-resistant forms of BCR-ABL, except the most refractory gatekeeper T315I mutation, which arises in more than 20% of CML patients^12^^,^^13^. The T315I mutation restrains the binding of first and second-generation BCR-ABL inhibitors to the ABL catalytic domain by either a direct steric hindrance or stabilising the active kinase conformation, which renders the design of new inhibitors targeting the open and active conformation of the T315I mutant as a major challenge^14^.

**Figure 1.**
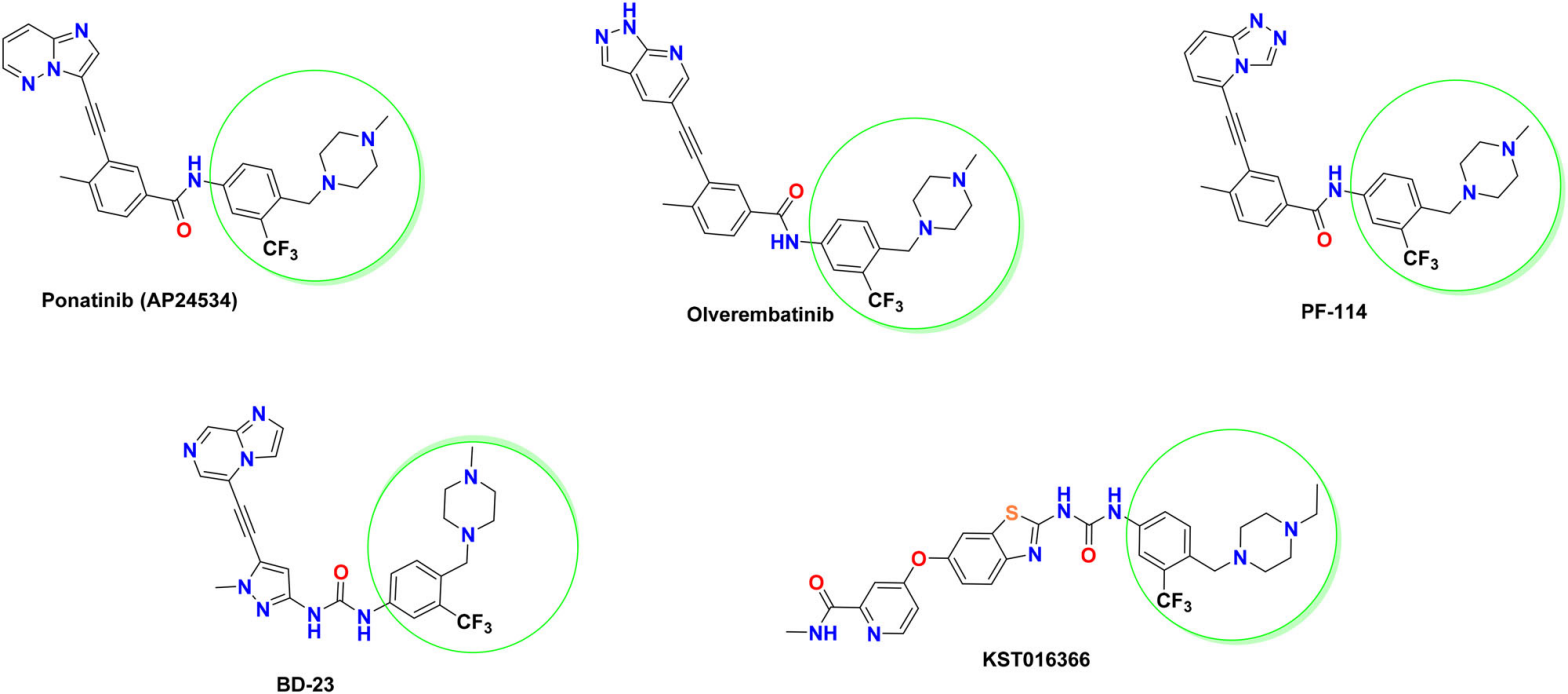
Representative examples of potent BCR-ABL^T315I^ inhibitors bearing 4-((4-methyl(ethyl)piperazin-1-yl) methyl)-3-(trifluoromethyl)phenyl moiety.

Ponatinib is the first FDA-approved third-generation BCR-ABL inhibitor for CML (Figure 1), which displayed excellent potency to inhibit BCR-ABL^WT^, BCR-ABL^T315I^, and other clinically significant ABL mutants, like Q252H, Y253H, M351T, and H396P^15^^,^^16^. Most recently, asciminib has been approved by FDA in October 2021 for the treatment of CML^17^. Being different from the existing tyrosine kinase inhibitors (TKIs), asciminib is the first allosteric BCR-ABL1 inhibitor specifically targeting the ABL1 myristoyl pocket (STAMP)^18^. It has demonstrated clinical activity in heavily pre-treated CML patients with or without mutations, with promising efficacy in patients with T315I mutation^19^. In addition, a number of structurally diverse BCR-ABL^T315I^ inhibitors have been identified (Figure 1). Replacing imidazo[1,2-*b*]pyridazine, the hinge binding motif, of ponatinib with other bioisosteric heterocycles afforded several promising BCR-ABL^T315I^ inhibitors. Among them, olverembatinib (HQP1351)^20^^,^^21^, and PF-114^22^^,^^23^ were developed as potent BCR-ABL^T315I^ inhibitors with better safety profile than ponatinib. Apart from these diarylamide acetylene-containing inhibitors, certain heteroaryl ureides such as BD-23^24^ and KST016366^25^ (Figure 1) were also reported as potent ABL^T315I^ inhibitors. As highlighted in Figure 1, the presence of 4-((4-methyl(ethyl)piperazin-1-yl) methyl)-3-(trifluoromethyl)phenyl moiety is a common prerequisite structural feature in the above mentioned BCR-ABL^T315I^ inhibitors. This privileged moiety serves as a tail, where the terminal nitrogen of piperazinyl methylene is predicted to be protonated at physiologic pH and then can engage in a hydrogen bond (HB) with the carbonyl oxygen of residue Ile360 in the activation loop of BCR-ABL^T315I^^15^.

**Figure 2.**
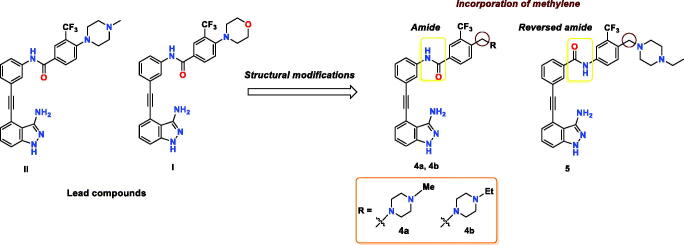
The chemical structure of lead compounds **II** and **I**, and the newly designed target compounds.

Recently, our group reported a series of 3-aminoindazole based ABL inhibitors^26^, where compound **I** (Figure 2) was identified as a potent ABL^WT^ inhibitor (IC_50_ = 4.6 nM) with excellent anti-leukemic activity against K-562 cell line (GI_50_ < 10 nM). However, compound **I** elicited moderate potency against the imatinib-resistant T315I mutant (IC_50_ = 227 nM). As disclosed from the SAR study of this series, the nature of the substituent installed on the 3-trifluoromethylphenyl ring greatly affects the ABL^T315I^ inhibitory potency^26^. In view of this finding, along with the substantial role of 4-((4-methyl(ethyl)piperazin-1-yl) methyl)-3-(trifluoromethyl)phenyl moiety in achieving favourable activity towards ABL^T315I^, we were motivated to further improve the activity of **I** against BCR-ABL^T315I^ as a part of our ongoing endeavours to discover potent BCR-ABL^T315I^ inhibitors^27^^,^^28^. In the current study, we designed a concise library of three indazoles **4a**, **4b**, and **5**, where the amide bond of **I** was conserved, as in **4a**, **4b**, while it was reversed in compound **5** (Figure 2). Most importantly, the 4-morpholino moiety of **I** was replaced by the privileged 4-((4-methyl(ethyl)piperazin-1-yl) methylene in all three compounds. To investigate the impact of methylene insertion between the terminal phenyl and substituted piperazine on BCR-ABL^T315I^ potency, we compared the activity of compounds **4a**, **4b**, and **5** with compound **II**, which lacks such methylene group (Figure 2). The newly designed compounds were synthesised, and evaluated against both ABL^WT^ and ABL^T315I^, and the most potent member **5** was further profiled against a set of clinically important ABL mutants.

## Results and discussion

### Chemistry

**Scheme 1.**
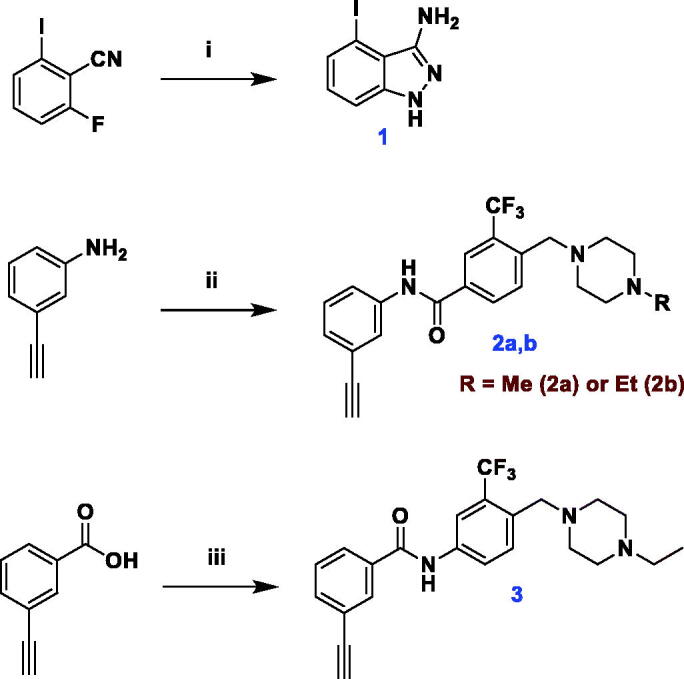
Synthesis of 3-aminoindazole **1** and diarylamides **2a, 2b** and **3**. Reagents and reaction conditions: **i**) 2-fluoro-6-iodobenzonitrile, *n*-BuOH, hydrazine hydrate, 110 °C, 2 h, 99%; **ii**) 4-((4-methyl(ethyl)piperazin-1-yl)methyl)-3-(trifluoromethyl)benzoic acid, HATU, DIPEA, DMF, rt, overnight, 74% (**2a**), 66% (**2b**); **iii**) 4-((4-ethylpiperazin-1-yl)methyl)-3-(trifluoromethyl)aniline, HATU, DIPEA, DMF, 60 °C, 3 h, 31%.

The key precursor for synthesis of target compounds, 4-iodoindazol-3-yl amine **1**, as well as diarylamides intermediates **2a**, **2b** and **3** were prepared as depicted in Scheme 1. Treatment of 2-fluoro-6-iodobenzonitrile with hydrazine hydrate in *n*-butanol at 110 °C furnished the 3-aminoindazole **1**^29^. Amide coupling of 3-ethynylaniline or 3-ethylnylbenzoice acid with the proper 4-((4-methyl(ethyl)piperazin-1-yl)methyl)-3-(trifluoromethyl)benzoic acid or aniline was accomplished using hexafluorophosphate azabenzotriazole tetramethyl uronium (HATU) and *N*,*N*-diisopropylethylamine (DIPEA) in DMF to afford the corresponding diarylamides.

**Scheme 2.**
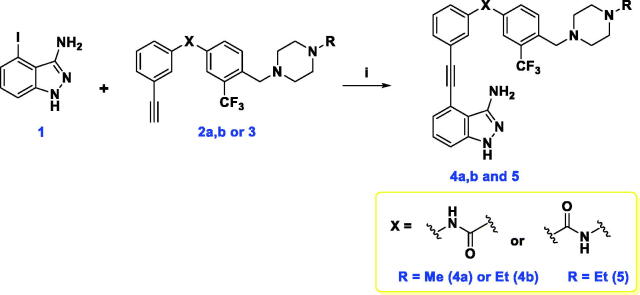
Synthesis of compounds **4a**, **4b** and **5**. Reagents and reaction conditions: **i**) PdCl_2_(PPh_3_)_2_, CuI, Et_3_N, DMF, 85 °C, dark, overnight, 19–27%.

Sonogashira coupling of **1** with the proper acetylene diarylamide **2a**, **2b** or **3** was carried out utilising PdCl_2_(PPh_3_)_2_ and CuI as catalysts in Et_3_N/DMF (1:1) at 85 °C to yield the diarylamide tethered indazoles **4a**, **4b** and **5** as illustrated in Scheme 2.

### *In vitro* biochemical kinase screening

#### ABL^WT^ and ABL^T315I^ kinase evaluations

Compounds **4a**, **4b** and **5** were tested against the native BCR-ABL^WT^ and its clinically relevant imatinib-resistant mutant BCR-ABL^T315I^ to determine their IC_50_ values, using ponatinib as a reference compound, and the pan kinase inhibitor staurosporine, as a positive control (Table 1). As disclosed from the data, compounds **4a** and **5** showed > 300 folds superior potency against BCR-ABL^WT^ rather the lead indazole **II** with IC_50_ values less than 0.51 nM. Such finding points out the remarkable role of methylene linker incorporated between the terminal phenyl and substituted piperazine in improving BCR-ABL^WT^ inhibition, which may stem from the ability of extended piperazine moiety to form tight interactions with the allosteric site of ABL. The *N*-methylpiperazine amide **4a** elicited slightly better inhibitory activity (BCR-ABL^T315I^ IC_50_ = 96 nM) than its corresponding *N*-ethylpiperazine analog **4b** (BCR-ABL^T315I^ IC_50_ = 142 nM). Meanwhile, both **4a** and **4b** surpassed the potency of lead indazoles **II** and **I** towards BCR-ABL^T315I^ with 8–11, and about two times, respectively. Interestingly, compound **5**, the reversed amide of **4b**, showed robust potency towards BCR-ABL^T315I^ with IC_50_ value of 9 nM, being 16 and 25 folds more potent than **4b** and lead **I**, respectively. Accordingly, it could be inferred that both the amide bond direction, the DFG binding motif, and the substitution of distal phenyl with ethylpiperazine methylene moiety at *para*-position contribute significantly in achieving optimal ABL^T315I^ inhibition. Compared with ponatinib, compound **5** showed better potency against ABL^WT^, and relatively lower potency for BCR-ABL^T315I^, yet both candidates possess single-digit nanomolar IC_50_ values. While comparing compound **5** with two ponatinib analogs, olverembatinib (HQP1351)^20^ and PF-114^22^, **5** showed comparable activity against BCR-ABL^WT^ and lower potency over BCR-ABL^T315I^. On the other hand, compound **5** (**AKE-72**) exerted superior potency than the ureidobenzothiazole KST016366 (BCR-ABL^WT^ and BCR-ABL^T315I^ IC_50_ = 53 nM)^25^.

**Table 1.** IC_50_ values (nM) of compounds **II**, **I**, **4a**, **4b**, and **5** against BCR-ABL^WT^ and BCR-ABL^T315I^.^a,b^.

Compound No.	IC_50_ (nM)	
	BCR-ABL^WT^	BCR-ABL^T315I^
**I** ^b^	4.6 ± 0.6	227 ± 44
**II** ^b^	151	1120
**4a**	< 0.51	96 ± 1.47
**4b**	0.64 ± 0.0099	142 ± 18.9
**5**	< 0.51	9 ± 3.14
**Ponatinib**	0.51 ± 0.0021	1.28 ± 0.0001
**Staurosporine**	83.8 ± 5.1	42.3 ± 13.4

^a^Compounds were tested in a 10-dose duplicate IC_50_ mode with 3-fold serial dilution starting at 10, 20 µM or 100 µM, and the reactions were carried out at 10 µM ATP.

^b^Data were retrieved from reference^26^.

Compound **5 (AKE-72)** was further profiled against a number of clinically relevant ABL mutants, including E255K, F317I, H396P, M351T, and Q252H (Table 2). This outcome confirmed the ability of compound **5** to potently suppress the activity of the clinically relevant ABL mutants in single-digit nanomolar IC_50_ values.

**Table 2.** *In vitro* enzymatic activity of **5 (AKE-72)** over wild-ABL and a panel of its clinically resistant mutants.

IC_50_ (nM)^a^				
ABL^E255K^	ABL^F317I^	ABL^H396P^	ABL^M351T^	ABL^Q252H^
8.98	3.12	< 1.02	3.44	3.88

^a^Compound **5** was tested in a 10-dose IC_50_ mode with 3-fold serial dilution starting at 20 μM, and the reactions were carried out at 10 μM ATP.

#### Kinase profile of 5

**Figure 3.**
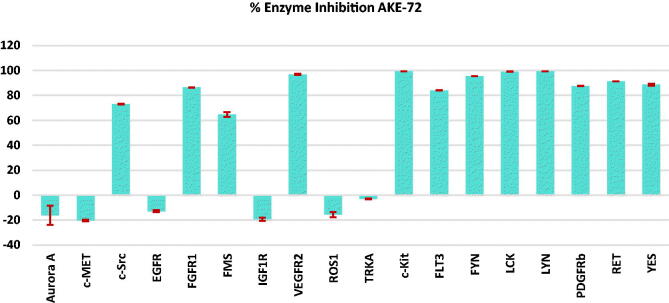
Enzyme inhibition percentage (relative to DMSO controls) of compound **5** (0.05 µM) against a panel of 18 kinases.

Furthermore, the most potent indazole member **5** was tested, at 50 nM concentration, over a panel of 18 major oncogenic kinases including the commonly kinases inhibited by ponatinib. As depicted in Figure 3, compound **5** showed significant inhibition against c-Kit, FGFR1, FLT3, FYN, LCK, LYN, PDGFR_β_, RET, VEGFR2, and YES kinases with (83.9–99.3% inhibition). While **5** displayed moderate suppressive activity (64.5–73% inhibition) over c-Src and FMS kinases, it exerted no inhibition against the other investigated kinases. This inhibition pattern presents compound **5** (**AKE-72**) as a multi-tyrosine kinase inhibitor in a similar fashion to that observed with ponatinib^16^^,^^30^.

### *In vitro* evaluation of the anti-leukemic activity by SRB assay

**Figure 4.**
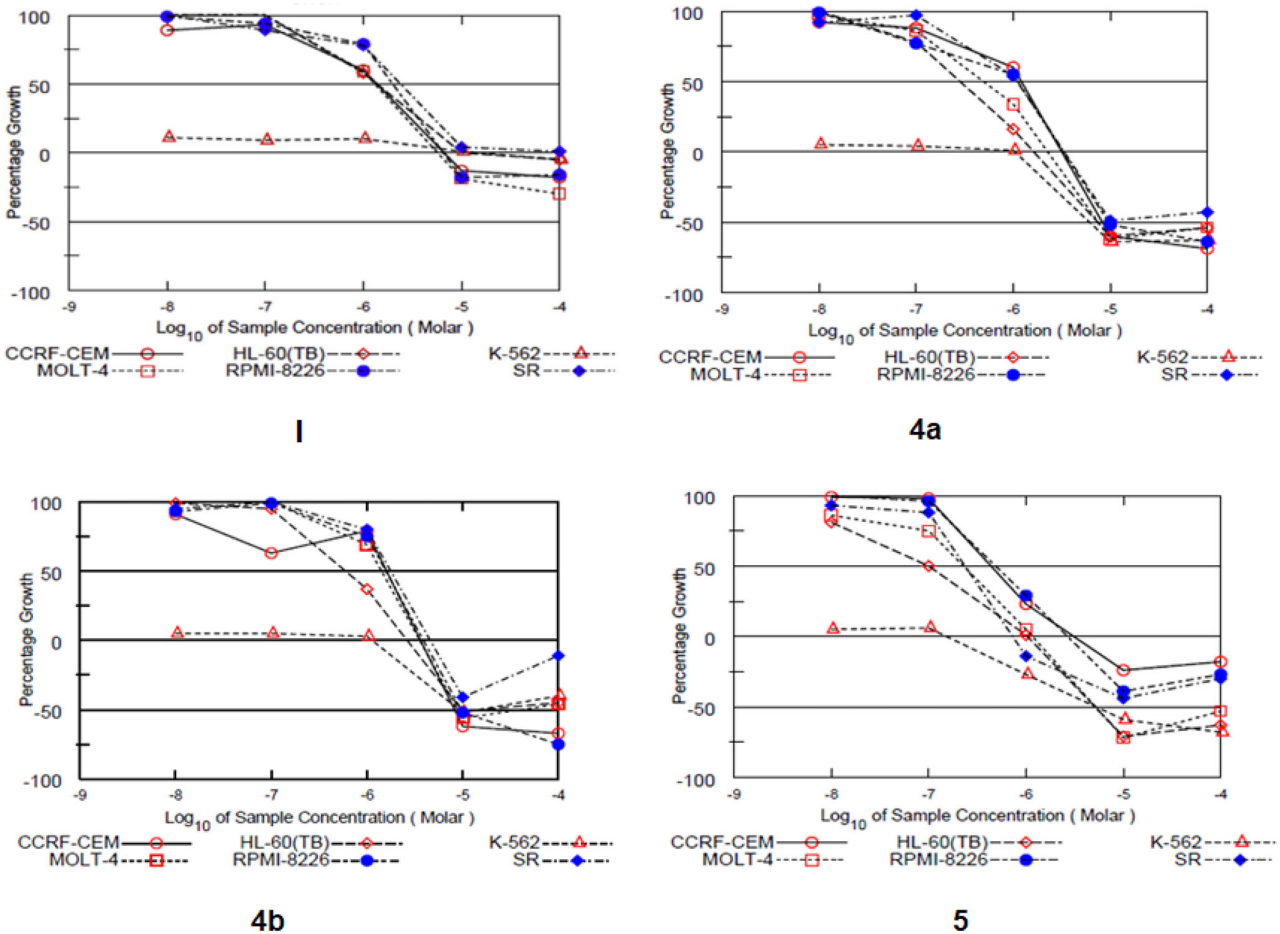
Dose-response curves of compounds **I**, **4a**, **4b**, and **5** against a panel of six human leukaemia cell lines.

Motivated by the promising biochemical assay data, the anti-leukemic activities of compounds **4a**, **4b**, and **5** were evaluated against a panel of six human leukaemia cell lines -using sulforhodamine B (SRB) assay- at National Cancer Institute (NCI, Developmental Therapeutics Program, Bethesda, MD, USA). The three compounds were tested initially at a single dose (10 μM), and their corresponding GI_50_ values were further determined by advancing to five-dose testing mode (Figure 4), and compared with the lead indazole **I** (Table 3).

**Table 3.** The antiproliferative activities (% Growth inhibition (GI) at 10 µM and GI_50_ (µM)) of compounds **I**, **4a**, **4b**, and **5** against a panel of six human leukaemia cell lines.^a,b^.

Leukaemia	**I**		**4a**		**4b**		**5**	
cell lines	GI %	GI_50_	GI %	GI_50_	GI %	GI_50_	GI %	GI_50_
CCRF-CEM	9.49	1.36	L	1.22	96.4	1.60	L	0.436
HL-60(TB)	99.01	1.40	NT	0.278	NT	0.593	L	0.0979
K-562	97.96	**< 0.01**	NT	**< 0.01**	NT	**< 1.00**	L	**< 0.01**
MOLT-4	92.33	1.31	NT	0.494	NT	1.42	L	0.227
RPMI-8226	L	1.98	L	1.22	L	1.59	L	0.488
SR	87.42	2.40	NT	1.10	NT	1.78	L	0.238

^a^L: lethal effect (% GI > 100), NT: not tested, NI: no inhibition. ^b^Bold figures refer to GI_50_ less than 10 nM.

The single-dose assay results pointed out the sound antiproliferative activity of compound **5** (GI > 100%) against all tested leukaemia cell lines. Most importantly, the five dose testing findings highlighted the remarkable selective anti-leukemic activities of all three compounds towards the ABL overexpressing leukaemia cell K562 with half-maximal growth inhibition concentration (GI_50_) values of less than 10 nM. This observation emphasises the excellent ability of compounds **4a**, **4b**, and **5** to suppress ABL activity on the cellular level. Over other leukaemia cells, both amides **4a** and **4b** showed comparable antiproliferative activity, with a special preference for HL-60 and MOLT-4 cell lines. For example, compound **4a** displayed GI_50_ values of 0.278 and 0.494 µM against HL-60 and MOLT-4 cells, respectively. Of special importance, the indazole reversed amide **5 (AKE-72)** elicited the highest cellular potency over all cell lines with sub-micromolar GI_50_ values. However, based on the activity order, **5** possessed particular selectivity towards the K-562 cell line. The noticed differential cellular potency of compounds **4a**, **4b**, and **5** may refer to their potential enzymatic selectivity towards ABL rather than other cancer-related kinases.

Apart from the potency parameter GI_50_, two additional measures; TGI (total growth inhibition) and LC_50_ (half-maximal lethal concentration) were calculated from the five dose assay and used as efficacy indices (Table 4). All three compounds exerted selective superior efficacy against K-562 cell line rather the lead indazole **I**. Particularly, indazole **5** induced total growth inhibition of K-562 cells at 154 nM, with 87-fold improved efficacy than **I** (TGI = 13500 nM). In addition, compound **5** triggered 50% cytotoxic lethal activity towards K-562 cells at 5.25 µM, while the lead **I** could not achieve same effect up to 100 µM.

**Table 4.** TGI and LC_50_ (µM) of compounds **I**, **4a**, **4b** and **5** against a panel of six human leukaemia cell lines.

Leukaemia	**I**		**4a**		**4b**		**5**	
cell lines	TGI	LC_50_	TGI	LC_50_	TGI	LC_50_	TGI	LC_50_
CCRF-CEM	6.58	>100	3.18	8.30	3.62	8.16	3.04	>100
HL-60(TB)	10.5	>100	1.6	7.33	2.61	ND^a^	1.04	5.17
K-562	13.5	>100	1.02	6.03	1.14	ND^a^	0.154	5.25
MOLT-4	5.71	>100	2.28	7.57	3.57	ND^a^	1.16	5.13
RPMI-8226	6.50	>100	3.26	9.52	3.92	9.71	2.66	>100
SR	>100	>100	3.35	>100	4.59	>100	0.732	>100

^a^ND: not determined.

### Further cell-based evaluation of the antiproliferative activity of compound 5 (AKE-72)

Further cellular proliferation assays were carried out for compound **5** (**AKE-72**) with normal RAW264.7 macrophage cells, Ba/F3 mouse pro-B cells and Ba/F3 cells expressing native BCR-ABL or BCR-ABL^T315I^ kinases following the reported protocol^31^ and using ponatinib as a reference compound (Table 5). **AKE-72** potently suppressed the proliferation of Ba/F3 cells expressing wild-type BCR-ABL (GI_50_ = 9.6 nM). In addition, the BCR-ABL^T315I^ mutant showed sensitivity to **AKE-72** (GI_50_ = 290 nM). Growth of Ba/F3 mouse pro-B cells and normal RAW264.7 macrophage cells was inhibited only at significantly higher GI_50_ of 4001 nM and 6982 nM, respectively. These cellular outcomes underscore the favourable differential selectivity of compound **AKE-72** for inhibiting BCR-ABL-dependent cells with minimal impact on normal cells. We also tested **AKE-72** against BCR-ABL-positive and BCR-ABL-negative cell lines. Compound **AKE-72** strongly inhibited the growth of K562 (BCR-ABL-positive, GI_50_ = 5.9 nM), however, had no significant activity against U937 (BCR-ABL-negative, GI_50_ = 1900 nM) leukaemia cell line (Table 5).

**Table 5.** GI_50_ (µM) of compound **5 (AKE-72)** and ponatinib against RAW264.7, Ba/F3 (Ba/F3 mouse pro-B cells), Ba/F3 (Bcr-Abl), Ba/F3 (Bcr-Abl^T315I^), K562, and U937 cells.^a^

Cell line	GI_50_ (µM)	
	5 (AKE-72)	Ponatinib
RAW264.7	6.982 ± 0.402	2.881 ± 0.153
Ba/F3 (mouse pro-B cell)	4.001 ± 1.166	0.708 ± 0.037
Ba/F3 (Bcr-Abl)	0.0096 ± 0.002	0.0021 ± 0.0001
Ba/F3 (Bcr-Abl^T315I^)	0.290 ± 0.036	0.015 ± 0.005
K562 (Bcr-Abl positive)	0.0059 ± 0.000	0.0019 ± 0.0002
U937 (Bcr-Abl negative)	1.900 ± 0.061	0.560 ± 0.125

^a^The presented data are the average of three independent experiments with standard deviations.

### CYP450 assays and ADME-Tox predictions

Azole derivatives are well known for their ability to inhibit cytochrome P450 isozymes. Since our target compounds feature azole moiety as a part of the indazole scaffold, we tested the affinity of **AKE-72** against four of the major cytochrome P450 isozymes at 10 µM; CYP1A2, CYP2D6, CYP2C9, and CYP3A4 (Table 6). As disclosed from the results, compound **5 (AKE-72)** showed weak inhibitory activity towards both CYP1A2 and CYP2D6 with 9.88% and 13.92% inhibition (IC_50_ > 10 µM), being less potent than furafylline and ketoconazole, respectively. In contrast, **5** elicited significant affinity against CYP2C9 (50.67% inhibition) and CYP3A4 (90.57% inhibition). The strong inhibitory effect with CYP3A4 is a common feature for many tyrosine kinase inhibitors, including Ponatinib^32^. Therefore, **AKE-72** has a reasonable CYP450-profile, which could be further improved in our next plan to offer merit for avoiding undesirable drug-drug interactions.

**Table 6.** CYP inhibition profile for compound **5** and ketoconazole/furafylline as reference standards.^a^

Compound No.	CYP inhibition % (IC_50_, µM)			
	CYP1A2	CYP2D6	CYP2C9	CYP3A4
**5**	9.88 ± 0.09 (> 10)	13.92 ± 0.06 (> 10)	50.67 ± 0.075 (NT)	96.57 ± 0.065 (NT)
**Ketoconazole**	–	(6.98)	(2.35)	(0.0017)
**Furafylline**	(0.519)	–	–	–

^a^Compound **5** was tested in a single-dose duplicate mode at a concentration of 10 μM, NT: not tested.

Furthermore, the pharmacokinetics ADME-Tox properties for compound **5** (**AKE-72**) were calculated by pkCSM, a web-based tool that can predict small-molecule pharmacokinetic and toxicity properties using graph-based signatures^33^. The ADME-Tox properties of compound **5** (data added to the supporting information) showed that it could be well absorbed from GIT. Compound **5** is predicted to be both a substrate and inhibitor for P-glycoprotein, which may offer the advantage of increasing bioavailability and efficacy, by inhibiting its own efflux from cells. In addition, compound **5** is predicted to be a non-substrate for hERG, indicating a low probability of causing *Torsade de points* cardiac adverse effects.

### Molecular docking study

**Figure 5.**
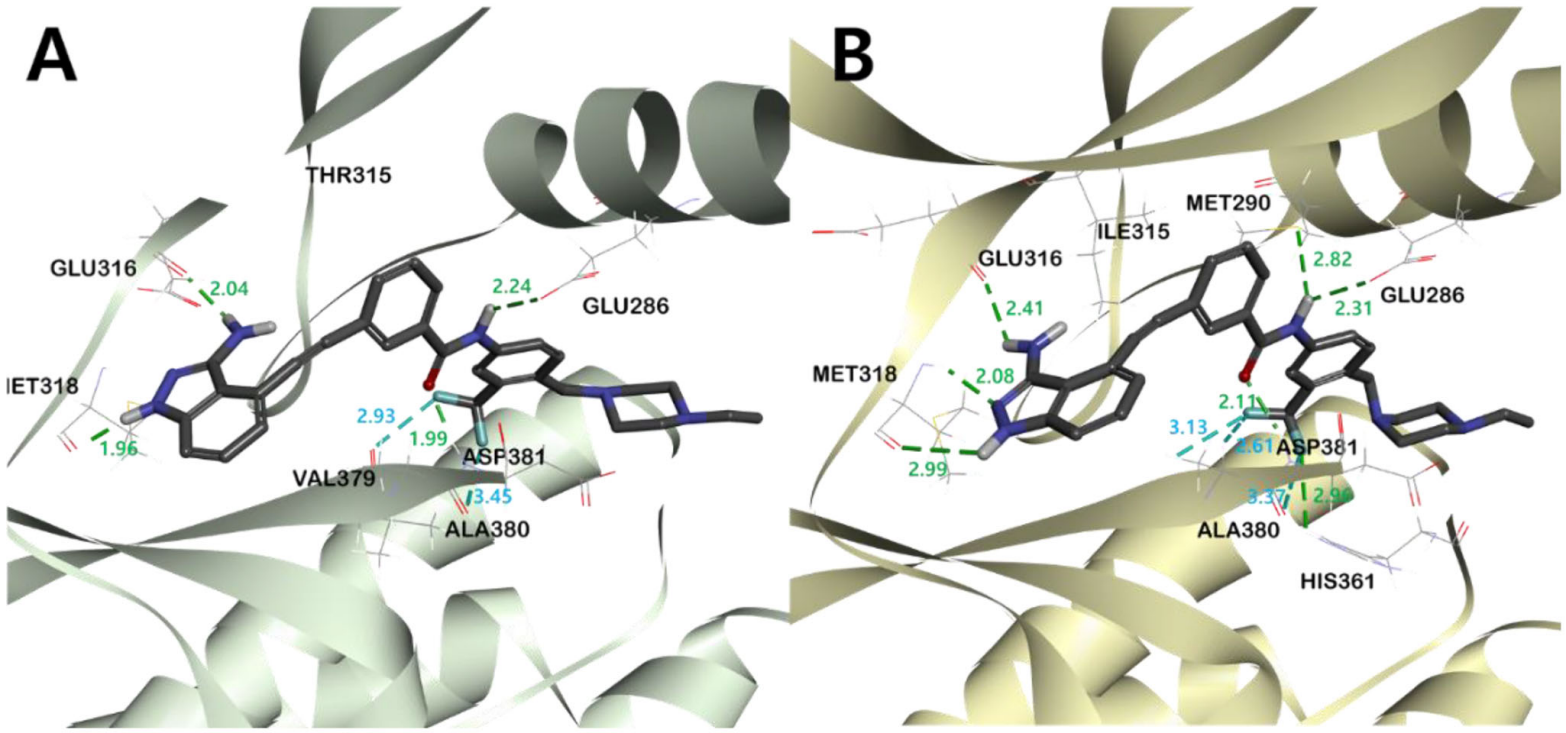
The binding mode of compound **5** in the kinase domain of (A) BCR-ABL^WT^ and (B) BCR-ABL^T315I^. For clarity, only the major residues with hydrogen bonding and halogen interactions are shown. Compound **5** is shown in the stick model, and surrounding key interaction residues were shown in the line model. Hydrogen bonding and halogen interactions are shown in dashes green and sky blue, respectively. The number near the dashes indicates the bonding distance in Å.

In order to rationalise the observed ABL kinase inhibitory results from a 3D structural perspective, the lead compounds **I** and **II**, and the newly designed derivatives **4a**, **4b**, and **5** were docked in the catalytic kinase domains of BCR-ABL^WT^ (PDB code: 3OXZ) and BCR-ABL^T315I^ (PDB code: 3OY3)^34^. The docking study revealed the existence of two major hydrogen bonds (HB) between all compounds and ABL kinases. One HB is between the carbonyl oxygen in the central amide bond of the inhibitor and the hydrogen in the amide side chain of Asp381, and the other is between the amidic hydrogen in the inhibitor and the carboxyl oxygen on the side chain of Glu286 (Figure 5, Figures S3 and S4). In addition, all inhibitors shared π-alkyl interactions between the phenyl group next to the indazole and Ile315 in BCR-ABL^T315I^. However, the main binding distance between compound **5** and the protein was relatively shorter in BCR-ABL^WT^ than in BCR-ABL^T315I^, suggesting that **5** binds more tightly to ABL^WT^ and exhibits a better inhibitory effect in ABL^WT^ (Figure S2-4).

**Figure 6.**
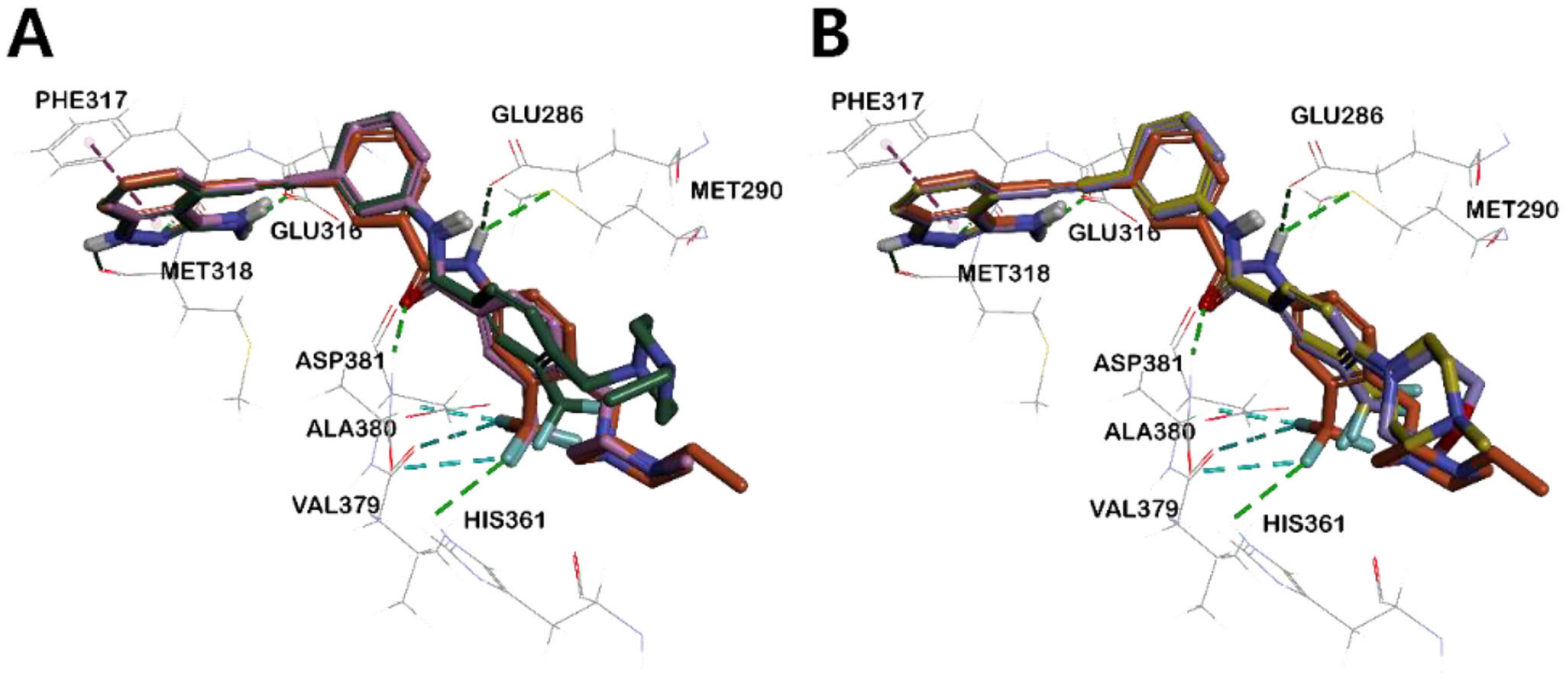
Superposed model of inhibitors inside BCR-ABL^T315I^ Kinase. (A) **5** (orange), **4a** (pink), and **4b** (green), (B) **5**, **I** (lavender), and **II** (yellow).

**Figure 7.**
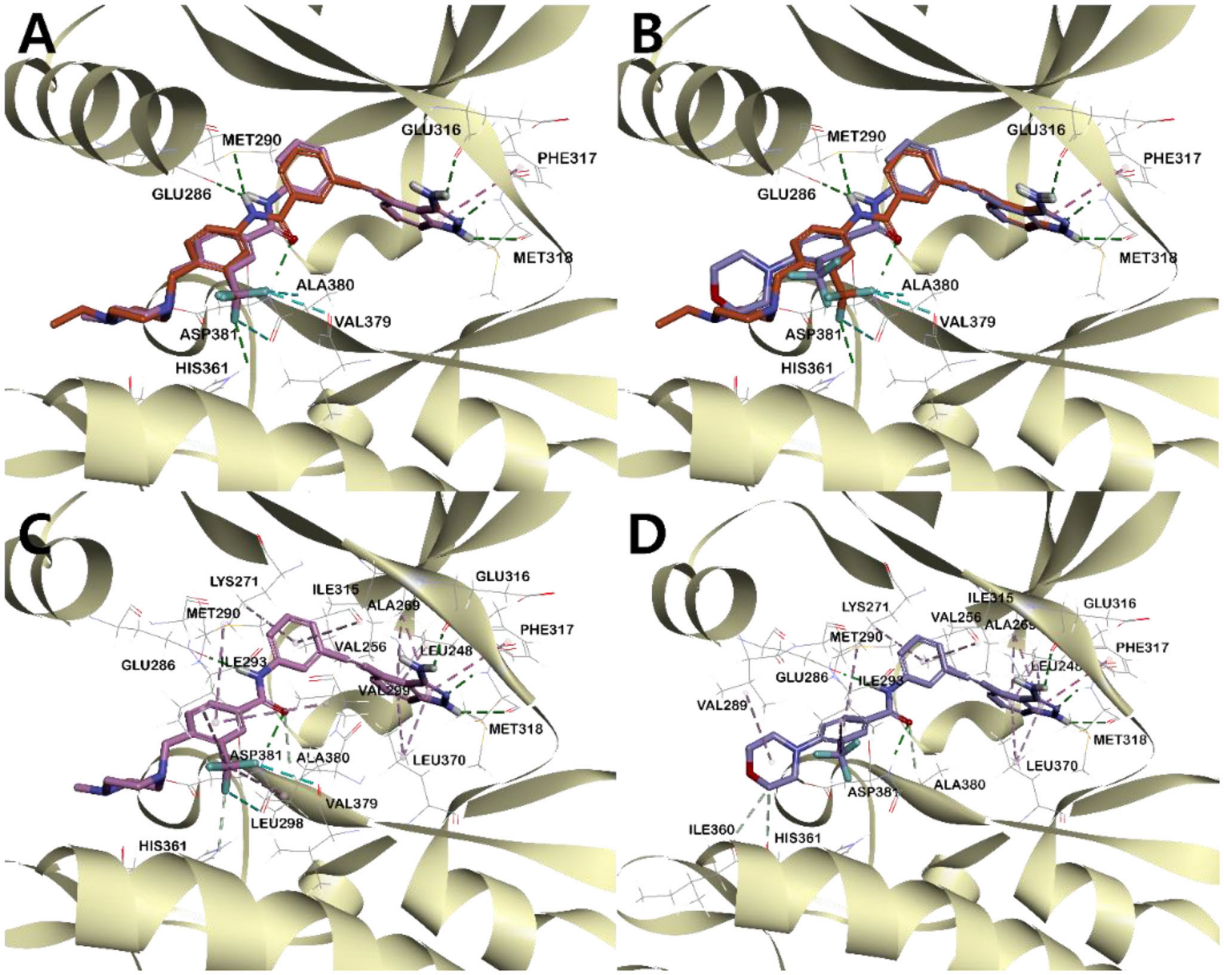
3D structural overlay of compounds (A) **5** (orange) and **4a** (pink), (B) **5** and **I** (lavender) in the ABL^T315I^ Kinase. Inhibitors are shown in stick model and surrounding key interaction residues were shown in line model. The key interaction mode between **5** and BCR-ABL^T315I^ indicated by a dash line. The binding mode of compounds (C) **4a**, and (D) **I** in the BCR-ABL^T315I^ Kinase. Various interactions are shown in dashes.

In all inhibitors, the indazole ring showed a very stable binding to both BCR-ABL^WT^ and BCR-ABL^T315I^ kinases. The indazole NH and 3-NH_2_ hydrogens were engaged in critical HB with the carboxyl group of Glu316 and Met318, respectively. The binding of indazole moiety to the ATP binding site of the ABL kinase was further stabilised by π- π interactions between Phe317 and the pyrazole moiety, as well as the hydrophobic interaction of the indazole moiety with hydrophobic residues such as Leu248, Val256, Ala269 and Leu370 (Figure 7(C,D), Figures S2–S4, S5C, S5D). Among all indazoles, compound **5** has the lowest binding energy and good inhibitory efficacy. The major difference between **5** and the other derivatives is that the central amide bond is reversed. This difference shifts the positions of the two phenyl groups slightly, resulting in different bonding modes and binding energies (Table S1). The phenyl group next to the stably bound indazole moiety exists on the same plane, but the plane of the opposite *m*-trifluoromethylphenyl group has different degrees of distortion. Compared to **5**, **4a** showed the smallest twist angle, and the *m*-trifluoromethylphenyl group planes of the remaining three inhibitors (**4b**, **I**, and **II**) were calculated to be bound to the protein by twisting at a relatively larger angle (Figure 6, Figure S6). The trifloromethyl group of compounds **5** and **4a**, which has the best potency against ABL^T315I^, was engaged in a halogen interaction with the Val379 and Ala380 residues, which is lacking in the other indazole derivatives (Figure 7(D) and Figure S5 (C-D)).

Importantly, in compound **5**, the *m*-trifluoromethylphenyl group and *N*-ethylpiperazine moiety are connected by a methylene linker. Such bulky piperazine moiety adopts a sterically more stable conformation that binds tightly with the protein, where the hydrogens in *N*-ethylpiperazine form carbon-hydrogen bonds with Ile360, His361, and Asp381, respectively. However, in the lead compound **I**, the morpholine moiety was directly bonded to trifluoromethylphenyl, thus both groups were positioned perpendicular to each other and bound to the protein in a form that reduced steric hindrance. Only one hydrogen of morpholine in compound **I** formed a carbon-hydrogen bond with Ile360 and His361, while alkyl interaction was noticed between morpholine and Val289. Overall, compound **I** showed fewer binding interactions with ABL compared to compound **5**. Therefore, the docking score of compound **I** was calculated to be lower than that of compound **5**, which is in agreement with the observed inhibitory effect (Figure 7(B,D), Figures S4A, S4E, CDocker interaction energy in Table 7). Similarly, in the case of compound **4a**, the presence of methylene linker allowed the bulky piperazine moiety to adopt a sterically more stable conformation with protein, and hydrogens in *N*-methylpiperazine could share carbon-hydrogen bonding with Ile360, His361, and Asp381, respectively. In contrast, in compound **II**, the phenyl group and bulky *N*-methyl piperazine were perpendicular to each other to reduce steric hindrance, which makes them bind to the ABL kinase in a different direction from that of *N*-methylpiperazine in **4a**. (Figure S4B, S4D, S5B, S5D). Compounds **4a** and **4b** possess methyl and ethyl substituents in their piperazine terminal NH, respectively. Rather than the effect of this, as suggested earlier, since the twist angle of the trifluoromethyl phenyl group increased in **4b**, it was unable to interact tightly with BCR-ABL^T315I^ and halogen (fluorine) and could not interact properly with other residues, as evidenced by the docking score (Figure S4).

**Table 7.** CDocker interaction energy of compounds **I**, **II**, **4a**, **4b**, and **5** with BCR-ABL^WT^ and BCR-ABL^T315I^ kinases.

Kinase	CDOCKER interaction energy (-kcal/mol)				
	I	II	4a	4b	5
BCR-ABL^WT^	56.8976	56.7543	64.2325	61.2787	65.0568
BCR-ABL^T315I^	60.854	60.3136	72.6524	64.9341	74.2774

## Experimental

The detailed experimental section is included in the supplementary data associated with this article.

## Conclusion

In this short communication, we report the design and synthesis of new indazole amides **4a**, **4b** and reversed amide **5** in attempt to improve the BCR-ABL inhibitory profile of our previously reported indazole lead compound **I**. The target compounds have been designed based on changing the morpholine motif of indazole **I** with the substantial (4-methyl(ethyl)piperazin-1-yl)methyl moiety, that exist in several potent BCR-ABL^T315I^ inhibitors. *In vitro* cell-free assays disclosed the excellent potency of all three compounds against BCR-ABL^WT^ as evident by their IC_50_ values of < 1 nM. Interestingly, compound **5** (**AKE-72**) exerted superior potency over the indazole **I** towards the most refractory T315I mutant with IC_50_ value of 9 nM. In addition, several forms of BCR-ABL mutants such as were greatly suppressed by **AKE-72** at single digit nanomolar IC_50_ values. Cellular screening of all compounds over a set of six human leukaemia cell lines, at NCI, pointed out their distinct and selective anti-leukemic potency towards K562 cell line, with GI_50_ less than 10 nM. In addition, **AKE-72** selectively inhibited the proliferation of Ba/F3 cells expressing native BCR-ABL or its T315I mutant. The putative binding mode of **AKE-72** in kinase domain of BCR-ABL^WT^, BCR-ABL^T315I^ was investigated by molecular docking study and revealed the important role of 4-((4-methyl(ethyl)piperazin-1-yl) methyl)-3-(trifluoromethyl)phenyl tail moiety in achieving tight binding within BCR-ABL kinase domain. Taken together, our current report offers compound **5 (AKE-72)** as a promising candidate for potential treatment of CML.

## Supplementary Material

Supplemental Material
