# Supplementary material for: Discovery of 3-((3-amino-1H-indazol-4-yl)ethynyl)-N-(4-((4-ethylpiperazin-1-yl)methyl)-3-(trifluoromethyl)phenyl)benzamide (AKE-72), a potent Pan-BCR-ABL inhibitor including the T315I gatekeeper resistant mutant
Source: J Enzyme Inhib Med Chem. 2023 Jul 20;38(1):2228515. doi: 10.1080/14756366.2023.2228515 (PMC10360995; doi:10.1080/14756366.2023.2228515)
Supplement: Supplemental Material [file IENZ_A_2228515_SM4012.pdf]

Supporting Information

Discovery of 3-((3-amino-*1H*-indazol-4-yl)ethynyl)-*N*-(4-((4-ethylpiperazin-1-yl)methyl)-3-(trifluoromethyl)phenyl)benzamide (AKE-72), a Potent Pan-BCR-ABL Inhibitor Including the T315I Gatekeeper resistant Mutant

Ashraf K. El-Damasy [a,b]*, Hyun Ji Kim [a], Jung Woo Park[c], Yunju Nam[d], Wooyoung Hur [d], Eun-Kyoung Bang [a], Gyochang Keum [a,e]*

[a] *Brain Science Institute, Korea Institute of Science and Technology (KIST), Seoul 02792, Republic of Korea*
[b]*Department of Medicinal Chemistry, Faculty of Pharmacy, Mansoura University, Mansoura 35516, Egypt*
[c]*Supercomputing Application Center, Div. of National Supercomputing, Korea Institute of Science and Technology Information, 245, Daehak-ro, Yuseong-gu, Daejeon 34141, Republic of Korea*
[d] *Medicinal Materials Research Center, Korea Institute of Science and Technology (KIST), 5 Hwarangro 14 Gil, Seongbuk-gu, Seoul 02792, Republic of Korea*
[e]*Division of Bio-Medical Science & Technology, KIST School, Korea University of Science and Technology (UST), Seoul 02792, Republic of Korea*

Corresponding author:
Ashraf K. El-Damasy, E-mail: ashraf.el-damasy@kist.re.kr, ph_karem2000@mans.edu.eg
Gyochang Keum, E-mail: gkeum@kist.re.kr

Contents **Pages**

1) Experimental section	2–4
2) ^{1}H NMR and ^{13}C NMR spectra	5–7
3) HRMS charts	8–10
4) ADME-Tox prediction by pkCSM	11
5) Molecular docking figures	12–14
6) References	15

1) Experimental section

1. General

All reactions and manipulations were performed utilizing standard Schlenk techniques. All solvents and reagents were commercially purchased and were utilized without further purification. The reaction progress was monitored on TLC plate (Merck, silica gel 60 F_{254}). Flash column chromatography was carried out using silica gel (Merck, 230–400 mesh) and the eluent solvents are indicated as volume-to-volume ratios. ^{1}H and ^{13}C NMR spectra were recorded on a Bruker Avance 400 MHz spectrometer, using the suitABLe deuterated solvents, as noted. Chemical shifts (δ) are reported in parts per million (ppm) upfield from tetramethylsilane (TMS) as internal standard. s, d, t, and m refer to singlet, doublet, triplet and multiplet, respectively, and coupling constants (*J*) are reported in hertz (Hz). High resolution mass spectra (HRMS) were recorded on Waters SYNAPT G2 mass spectrometer ESI-TOF mode. The purity of all final compounds was > 95%, as evidenced by ^{1}H NMR charts. 4-((4-Methyl(ethyl)piperazin-1-yl)methyl)-3-(trifluoromethyl)benzoic acid [1], 4-((4-ethylpiperazin-1-yl)methyl)-3-(trifluoromethyl)aniline [2], and 4-iodo-*1H*-indazol-3-amine (**1**)[3] were synthesized adopting the reported procedure.

*1.2. General procedure for synthesis of acetylene diarylamides **2a** and **2b***

To a mixture of commercially available 3-ethynylaniline (1.0 eq.), 4-((4-methyl(ethyl)piperazin-1-yl)methyl)-3-(trifluoromethyl)benzoic acid (1.2 eq.) and HATU (3.0 eq.) in anhydrous DMF (5 mL), DIPEA (4.0 eq.) were added. The reaction mixture was stirred at room temperature overnight. The reaction mixture was quenched with distilled water, and extracted with ethyl acetate (20×3 mL). The combined organic layers were washed with brine solution, dried over anhydrous Na_2SO_4 and filtered. The solvent was evaporated under reduced pressure, and the residue was purified by silica gel flash column chromatography using proper eluent to afford compounds **2a** and **2b**.

*1.2.1. N-(3-ethynylphenyl)-4-((4-methylpiperazin-1-yl)methyl)-3-(trifluoromethyl)benzamide (**2a**)*
The compound was purified by silica gel flash column chromatography using (MeOH:DCM = 1:9, then switching to 3:17). Yellow solid (74%); ^{1}H NMR (400 MHz, $CDCl_3$-*d*) δ 8.10 (s, 1H), 8.01–7.95 (m, 3H), 7.75 (brs, 1H), 7.68 (dt, *J* = 7.7, 1.7 Hz, 1H), 7.34–7.27 (m, 2H), 3.71 (s, 2H), 3.08 (s, 1H), 2.52–2.47 (m, 8H), 2.30 (s, 3H); ^{13}C NMR (100 MHz, $CDCl_3$-*d*) δ 164.58, 142.59, 137.82, 133.49, 131.10, 130.38, 129.36 (d, *J* = 31 Hz), 129.35, 128.74, 125.41, 126.82 (q, *J* = 5.7 Hz), 123.93, 123.21, 121.07, 83.15, 77.89, 58.09, 55.33, 53.36, 46.20.

*1.2.2. 4-((4-ethylpiperazin-1-yl)methyl)-N-(3-ethynylphenyl)-3-(trifluoromethyl)benzamide (**2b**)*
The compound was purified by silica gel flash column chromatography using (MeOH:28% NH_4OH:EA, 0.5:1:98.5). Yellow solid (66%); ^{1}H NMR (400 MHz, $CDCl_3$-*d*) δ 8.09 (s, 1H), 8.00–7.92 (m, 3H), 7.75 (br. s, 1H), 7.68 (dt, *J* = 7.7, 1.8 Hz, 1H), 7.34–7.27 (m, 2H), 3.71 (s, 2H), 3.08 (s, 1H), 2.53–2.40 (m, 10H), 1.09 (t, *J* = 7.4 Hz, 3H); ^{13}C NMR (100 MHz, $CDCl_3$-*d*) δ 164.43, 142.49, 137.63, 133.29, 130.96, 130.18, 129.19 (d, *J* = 31 Hz), 129.18, 128.58, 124.61 (q, *J* = 5.8 Hz), 123.88 (d, *J* = 273 Hz), 123.76, 123.05, 120.89, 82.98, 77.73, 57.97, 53.27, 52.87, 52.32, 12.04.

*1.3. N-(4-((4-ethylpiperazin-1-yl)methyl)-3-(trifluoromethyl)phenyl)-3-ethynylbenzamide (**3**)*
To a mixture of 3-ethynylbenzoic acid (0.111 mg, 0.761 mmol), 4-((4-ethylpiperazin-1-yl)methyl)-3-(trifluoromethyl)aniline (0.175 mg, 0.609 mmol) and HATU (579 mg, 1.52 mmol) in anhydrous DMF (2.5 mL), DIPEA (0.532 mL, 3.05 mmol) was added. The reaction mixture was stirred at 60 °C for 3 h, and saturated $NaHCO_3$ (aq.) was added for quenching. The aqueous layer was extracted with ethyl acetate (20 mL×3), and the combined organic layer were washed with brine solution, dried over anhydrous Na_2SO_4, and filtered. The solvent was evaporated under reduced pressure, and the residue was purified by silica gel flash column chromatography (MeOH:28% NH_4OH:EA, 2:1:97) to yield compound **3** as a yellow solid (31%). ^{1}H NMR (400 MHz, Acetone-d_6) δ 9.88 (s, 1H), 8.29 (s, 1H), 8.11–8.09 (m, 2H), 8.04 (d, *J* = 7.8 Hz, 1H), 7.81 (d, *J* = 8.5 Hz, 1H), 7.71 (d, *J* = 7.7 Hz, 1H), 7.57 (t, *J* = 7.8 Hz, 1H), 3.86 (br. s, 3H), 3.79 (s, 1H), 3.76 (s, 2H), 3.32 (br. s, 5H), 3.23 (q, *J* = 7.3 Hz, 2H), 1.35 (t, *J* = 7.3 Hz, 3H); ^{13}C NMR (100 MHz, Acetone-d_6) δ 165.69, 139.46 (d, *J* = 9.0 Hz), 136.09 (d,

J = 3.0 Hz), 135.82, 132.59, 132.39, 131.62, 129.84, 129.34 (d, *J* = 30 Hz), 128.91, 125.39 (d, *J* = 272 Hz), 124.24, 124.03, 123.55, 118.29 (q, *J* = 6.3 Hz), 83.29, 80.26, 57.93, 53.20, 53.10, 51.48, 10.33.

*1.4. General procedure for synthesis of target compounds **4a**,**4b** and **5***

To a solution of the proper acetylene diarylamide **2a, 2b** or **3** (1.0 eq.), and 3-amioindazole **1** (1.1 eq.) in anhydrous DMF (2.0 mL), bis(triphenylphosphine)palladium (II) chloride (0.02 eq.), and copper(I) iodide (0.04 eq.) was added. Next, triethylamine (2.0 mL) was added to the reaction mixture. The flask of reaction mixture was covered with aluminum foil and stirred at 85 °C overnight at dark. The reaction was cooled down to room temperature, quenched with saturated $NaHCO_3$ (aq.), and extracted with ethyl acetate (20 mL×3). The combined organic layers were washed with brine solution, dried over anhydrous Na_2SO_4 and filtered. The solvent was evaporated under reduced pressure, and the residue was purified by silica gel flash column chromatography using the proper eluent.

*1.4.1. N-(3-((3-amino-1H-indazol-4-yl)ethynyl)phenyl)-4-((4-methylpiperazin-1-yl)methyl)-3-(trifluoromethyl)benzamide (**4a**)*

The compound was purified by silica gel flash column chromatography using (MeOH:DCM, 1:19). Ivory solid (27%); ^{1}H NMR (400 MHz, DMSO-d_6) δ 11.81 (s, 1H), 10.58 (s, 1H), 8.27 (s, 1H), 8.25 (d, *J* = 8.2 Hz, 1H), 8.05 (s, 1H), 7.94 (d, *J* = 8.1 Hz, 1H), 7.85 (d, *J* = 8.0 Hz, 1H), 7.47 (t, *J* = 7.9 Hz, 1H), 7.39 (d, *J* = 7.7 Hz, 1H), 7.34 (d, *J* = 8.1 Hz, 1H), 7.27 (t, *J* = 7.7 Hz, 1H), 7.15 (d, *J* = 6.7 Hz, 1H), 5.14 (s, 2H), 3.69 (s, 2H), 2.42–2.36 (m, 8H), 2.17 (s, 3H); ^{13}C NMR (100 MHz, Methanol-d_4) δ 167.07, 150.29, 143.32, 142.93, 140.26, 135.16, 132.25, 132.16, 130.33, 130.01 (q, *J* = 31 Hz), 128.67, 128.42, 126.50 (q, *J* = 5.9 Hz), 125.66 (d, *J* = 272 Hz), 124.79, 124.66, 124.52, 122.71, 116.28, 113.84, 111.99, 94.46, 87.96, 59.07, 56.06, 53.80, 46.05; HRMS (ESI-TOF) m/z calcd for $C_{2I28}F_3N_6O$ [M+H]$^+$: 533.2277, found: 533.2286.

*1.4.2. N-(3-((3-amino-1H-indazol-4-yl)ethynyl)phenyl)-4-((4-ethylpiperazin-1-yl)methyl)-3-(trifluoromethyl)benzamide (**4b**)*

The compound was purified by silica gel flash column chromatography using (MeOH:DCM, 1:19). Yellow solid (57.7 mg, 19%); ^{1}H NMR (400 MHz, Methnol-d_4) δ 8.26 (s, 1H), 8.16 (d, *J* = 8.1 Hz), 8.01 (s, 1H), 7.96 (d, *J* = 8.1 Hz, 1H), 7.75 (dt, *J* = 7.3, 2.0 Hz, 1H), 7.43–7.37 (m, 2H), 7.34–7.28 (m, 2H), 7.17 (dd, *J* = 6.2, 1.5 Hz, 1H), 3.73 (s, 2H), 2.56–2.45 (m, 10H), 1.11 (t, *J* = 7.2 Hz, 3H); ^{13}C NMR (100 MHz, Methanol-d_4) δ 167.03, 150.22, 143.26, 142.84, 140.19, 135.10, 132.18, 132.11, 130.28, 129.95 (d, *J* = 31 Hz), 128.62, 128.36, 126.43 (d, *J* = 6.0 Hz), 125.60 (d, *J* = 272 Hz), 124.74, 124.60, 124.46, 124.24, 122.67, 116.21, 113.77, 111.94, 94.37, 87.88, 59.02, 53.71, 53.66, 53.27, 11.65; HRMS (ESI-TOF) m/z calcd for $C_{30}H_{30}F_3N_6O$ [M+H]$^+$: 547.2433, found: 547.2430.

*1.4.3. 3-((3-amino-1H-indazol-4-yl)ethynyl)-N-(4-((4-ethylpiperazin-1-yl)methyl)-3-(trifluoromethyl)phenyl)benzamide (**5**)*

The compound was purified by silica gel flash column chromatography using (MeOH:DCM, 3:97, then switching to MeOH: NH_4OH:DCM, 3:1:96). Yellow solid (21%); ^{1}H NMR (400 MHz, Acetone-d_6) δ 11.04 (br. S, 1H), 9.91 (s, 1H), 8.29 (s, 1H), 8.25 (s, 1H), 8.10 (d, *J* = 8.1 Hz, 1H), 8.07 (d, *J* = 8.6 Hz, 1H), 7.85 (d, *J* = 7.7 Hz, 1H), 7.81 (d, *J* = 8.6 Hz, 1H), 7.62 (t, *J* = 7.7 Hz, 1H), 7.41 (d, *J* = 8.4 Hz, 1H), 7.31 (t, *J* = 7.7 Hz, 1H), 7.21 (d, *J* = 7.0 Hz, 1H), 5.61 (s, 1H), 4.94 (br. s, 1H), 3.63 (s, 2H), 2.48 (br. s, 8H), 2.35 (q, *J* = 7.1 Hz, 2H), 1.02 (t, *J* = 7.1 Hz, 3H); ^{13}C NMR (100 MHz,) δ 164.88, 149.00, 142.01, 138.24 (d, *J* = 8.8 Hz); HRMS (ESI-TOF) m/z calculated for $C_{30}H_{30}F_3N_6O$ [M+H]+: 547.2433, found: 547.2438.

1.5. In vitro kinase screening

Reaction Biology Corporation (RBC) Kinase HotSpot^{SM} service was utilized for cell-free biochemical kinase evaluation of the target compounds according to the reported assay protocol [4].

1.6. Anti-leukemic screening at NCI

The anticancer screening of target compounds over a panel of six human leukemia cell lines was carried out using Sulforhodamine B (SRB) assay at the National Cancer Institute (NCI), Bethesda, Maryland, USA employing the standard protocol [5].

1.7. Further cell-based evaluation of the antiproliferative activity

Further cellular proliferation assays for compound **5** (**AKE-72**) were performed with normal RAW264.7 macrophage cells, parental Ba/F3 cells, Ba/F3 cells expressing native BCR-ABL or BCR-ABL^{T315I} kinases, K562, and U937 cell lines following the reported protocol [6].

1.8. Cytochrome P450 Assay

Cytochrome P450 inhibition assay of compound **5** was conducted at RBC using the Vivid CYP450 screening kits (by ThermoFisher) following the reported procedure [7].

1.9. Molecular docking

The molecular docking model of compound **I**, **II**, **4a**, **4b**, and **5** was constructed using the crystal structure of BCR-ABL^{WT} or BCR-ABL^{T315I} (PDB code: 3OXZ and 3OY3) [8] in its DFG-out conformation using Discovery Studio 2022 (DS). The protein structure of BCR-ABL^{WT} and BCR-ABL^{T315I} were prepared for docking by employing protocol "prepare protein" with and ligands were prepared through protonation at pH 7.4 and energy minimization. The binding site was defined based on ponatinib with BCR-ABL kinase domain. The ligands were docked at the defined binding sites using the CDOCKER algorithm.

2) ^{1}H NMR and ^{13}C NMR spectra

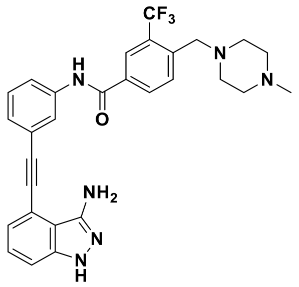

4a

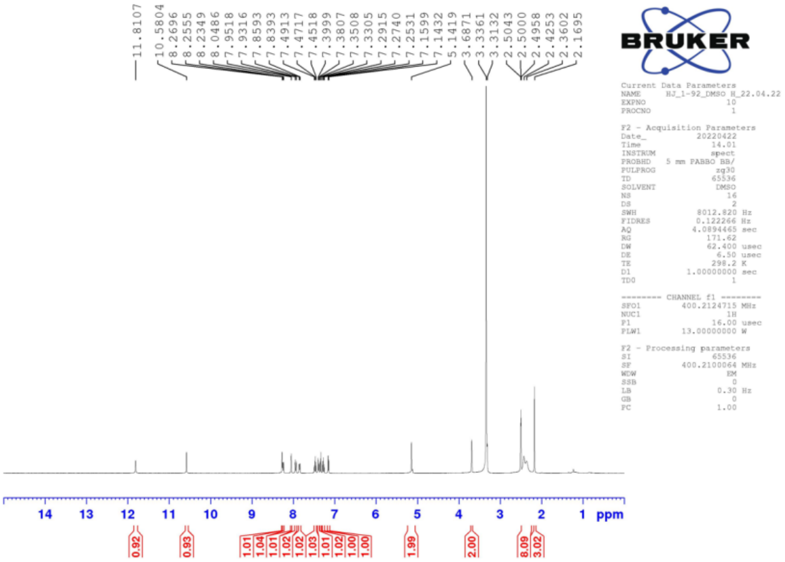

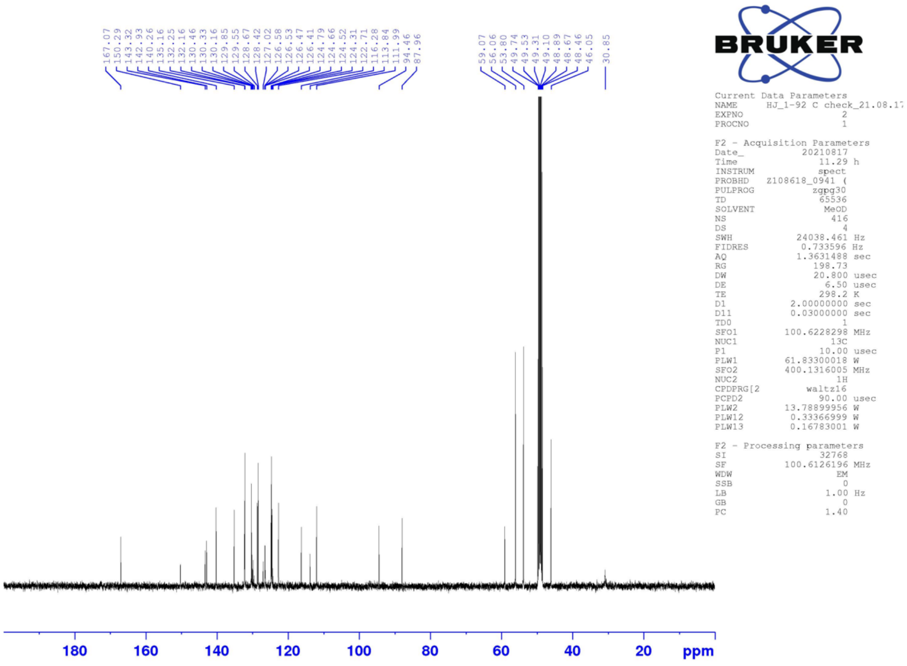

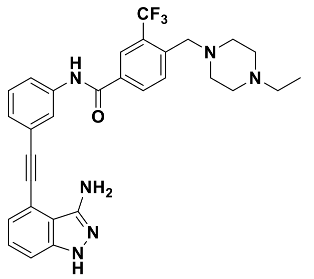

CF3
H
N
N
N
O
NH2
N
N
H

4b

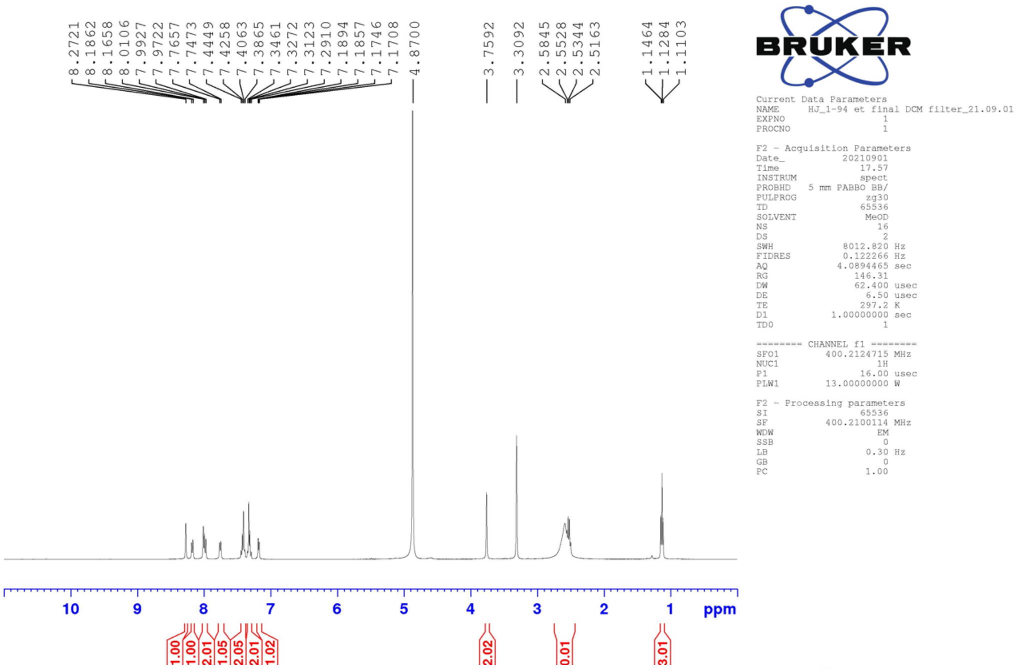

8.2721
8.1862
8.1658
8.0106
7.9927
7.9722
7.7657
7.7473
7.4449
7.4258
7.4063
7.3865
7.3461
7.3272
7.3123
7.2910
7.1894
7.1857
7.1746
7.1708
4.8700
3.7592
3.3092
2.5845
2.5528
2.5344
2.5163
1.1464
1.1284
1.1103
BRUKER
Current Data Parameters
NAME HJ_1-94 et final DCM filter_21.09.01
EXPNO 1
PROCNO 1
F2 - Acquisition Parameters
Date_ 20210901
Time 17.57
INSTRUM spect
PROBHD 5 mm PABBO BB/
PULPROG zg30
TD 65536
SOLVENT MeOD
NS 16
DS 2
SWH 8012.820 Hz
FIDRES 0.122266 Hz
AQ 4.0894465 sec
RG 146.31
DW 62.400 usec
DE 6.50 usec
TE 297.2 K
D1 1.00000000 sec
TD0 1
======= CHANNEL f1 =======
SFO1 400.2124715 MHz
NUC1 1H
P1 16.00 usec
PLW1 13.00000000 W
F2 - Processing parameters
SI 65536
SF 400.2100114 MHz
WDW EM
SSB 0
LB 0.30 Hz
GB 0
PC 1.00
10 9 8 7 6 5 4 3 2 1 ppm
1.00
1.00
2.01
1.05
2.05
2.01
1.02
2.02
10.01
3.01

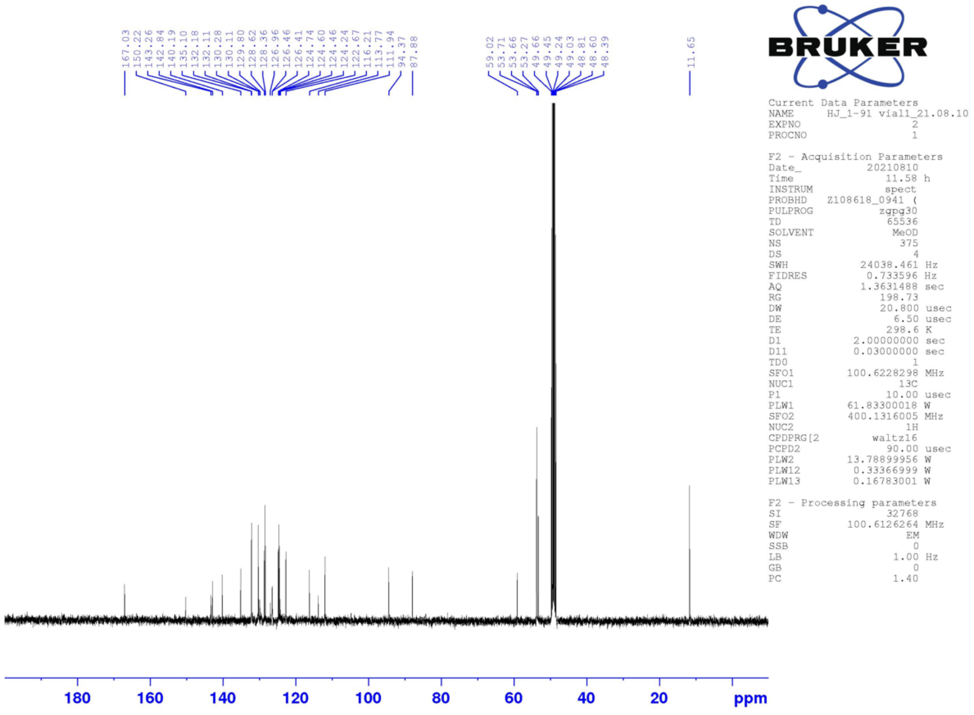

167.03
150.22
143.26
142.84
140.19
135.10
132.18
132.11
130.28
130.11
129.80
128.62
128.36
126.96
126.46
126.41
124.74
124.60
124.46
124.24
122.67
116.21
113.77
111.94
94.37
87.88
59.02
53.71
53.66
53.27
49.66
49.45
49.24
49.03
48.81
48.60
48.39
11.65
BRUKER
Current Data Parameters
NAME HJ_1-91 vial1_21.08.10
EXPNO 2
PROCNO 1
F2 - Acquisition Parameters
Date_ 20210810
Time 11.58 h
INSTRUM spect
PROBHD Z108618_0941 (
PULPROG zgpg30
TD 65536
SOLVENT MeOD
NS 375
DS 4
SWH 24038.461 Hz
FIDRES 0.733596 Hz
AQ 1.3631488 sec
RG 198.73
DW 20.800 usec
DE 6.50 usec
TE 298.6 K
D1 2.00000000 sec
D11 0.03000000 sec
TD0 1
SFO1 100.6228298 MHz
NUC1 13C
P1 10.00 usec
PLW1 61.83300018 W
SFO2 400.1316005 MHz
NUC2 1H
CPDPRG[2 waltz16
PCPD2 90.00 usec
PLW2 13.78899956 W
PLW12 0.33366999 W
PLW13 0.16783001 W
F2 - Processing parameters
SI 32768
SF 100.6126264 MHz
WDW EM
SSB 0
LB 1.00 Hz
GB 0
PC 1.40
180 160 140 120 100 80 60 40 20 ppm

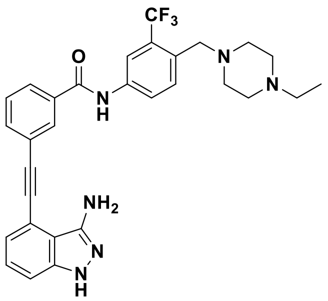
CF3
O
N
H
N
N
NH2
N
N
H

5

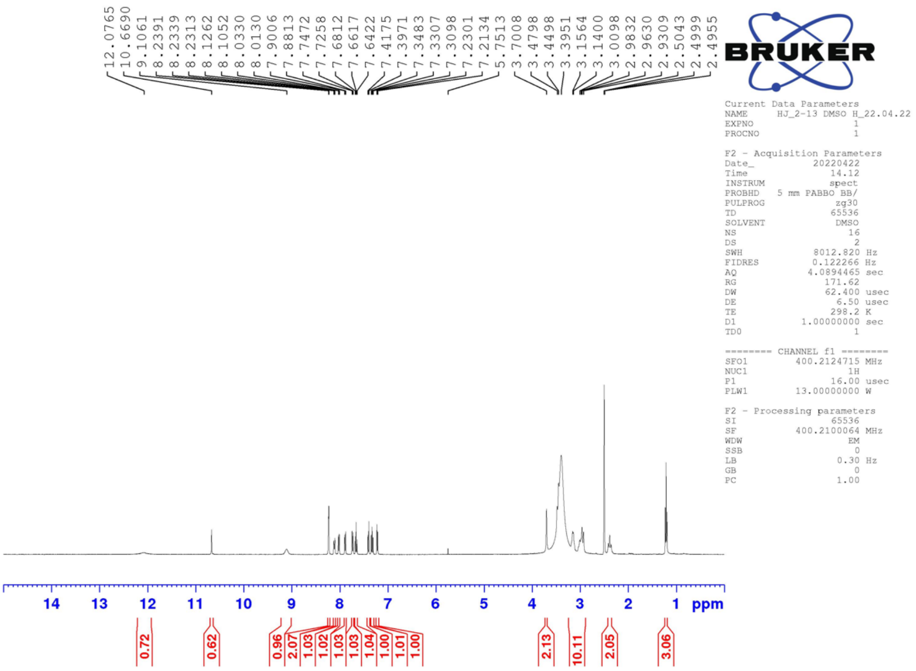
12.0765
10.6690
9.1061
8.2391
8.2339
8.2313
8.1262
8.1052
8.0330
8.0130
7.9006
7.8813
7.7472
7.7258
7.6812
7.6617
7.6422
7.4175
7.3971
7.3483
7.3307
7.3098
7.2301
7.2134
5.7513
3.7008
3.4798
3.4498
3.3951
3.1564
3.1400
3.0098
2.9832
2.9630
2.9309
2.5043
2.4999
2.4955
BRUKER
Current Data Parameters
NAME HJ_2-13 DMSO H_22.04.22
EXPNO 1
PROCNO 1
F2 - Acquisition Parameters
Date_ 20220422
Time 14.12
INSTRUM spect
PROBHD 5 mm PABBO BB/
PULPROG zg30
TD 65536
SOLVENT DMSO
NS 16
DS 2
SWH 8012.820 Hz
FIDRES 0.122266 Hz
AQ 4.0894465 sec
RG 171.62
DW 62.400 usec
DE 6.50 usec
TE 298.2 K
D1 1.00000000 sec
TD0 1
======= CHANNEL f1 =======
SFO1 400.2124715 MHz
NUC1 1H
P1 16.00 usec
PLW1 13.00000000 W
F2 - Processing parameters
SI 65536
SF 400.2100064 MHz
WDW EM
SSB 0
LB 0.30 Hz
GB 0
PC 1.00
14 13 12 11 10 9 8 7 6 5 4 3 2 1 ppm
0.72
0.62
0.96
2.07
1.03
1.02
1.03
1.03
1.04
1.00
1.01
1.00
2.13
10.11
2.05
3.06

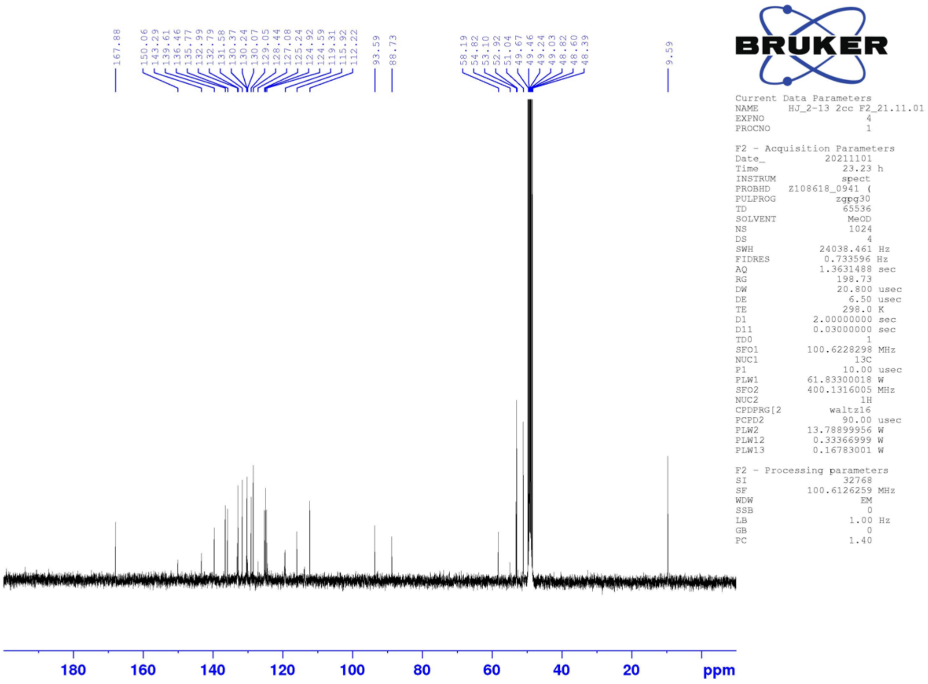
167.88
150.06
143.29
139.61
136.46
135.77
132.99
132.79
131.58
130.37
130.24
130.07
129.05
128.44
127.08
125.24
124.92
124.59
119.31
115.92
112.22
93.59
88.73
58.19
54.82
53.10
52.92
51.04
49.67
49.46
49.24
49.03
48.82
48.60
48.39
9.59
BRUKER
Current Data Parameters
NAME HJ_2-13 2cc F2_21.11.01
EXPNO 4
PROCNO 1
F2 - Acquisition Parameters
Date_ 20211101
Time 23.23 h
INSTRUM spect
PROBHD Z108618_0941 (
PULPROG zgpg30
TD 65536
SOLVENT MeOD
NS 1024
DS 4
SWH 24038.461 Hz
FIDRES 0.733596 Hz
AQ 1.3631488 sec
RG 198.73
DW 20.800 usec
DE 6.50 usec
TE 298.0 K
D1 2.00000000 sec
D11 0.03000000 sec
TD0 1
SFO1 100.6228298 MHz
NUC1 13C
P1 10.00 usec
PLW1 61.83300018 W
SFO2 400.1316005 MHz
NUC2 1H
CPDPRG[2 waltz16
PCPD2 90.00 usec
PLW2 13.78899956 W
PLW12 0.33366999 W
PLW13 0.16783001 W
F2 - Processing parameters
SI 32768
SF 100.6126259 MHz
WDW EM
SSB 0
LB 1.00 Hz
GB 0
PC 1.40
180 160 140 120 100 80 60 40 20 ppm

3) HRMS charts

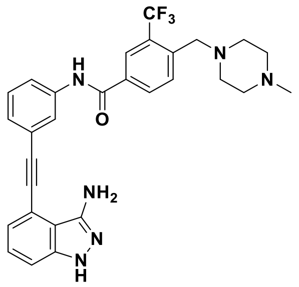

4a

$C_{29}H_{28}F_3N_6O$ $[M+H]^+$:

Calculated: 533.2277

Found: 533.2286

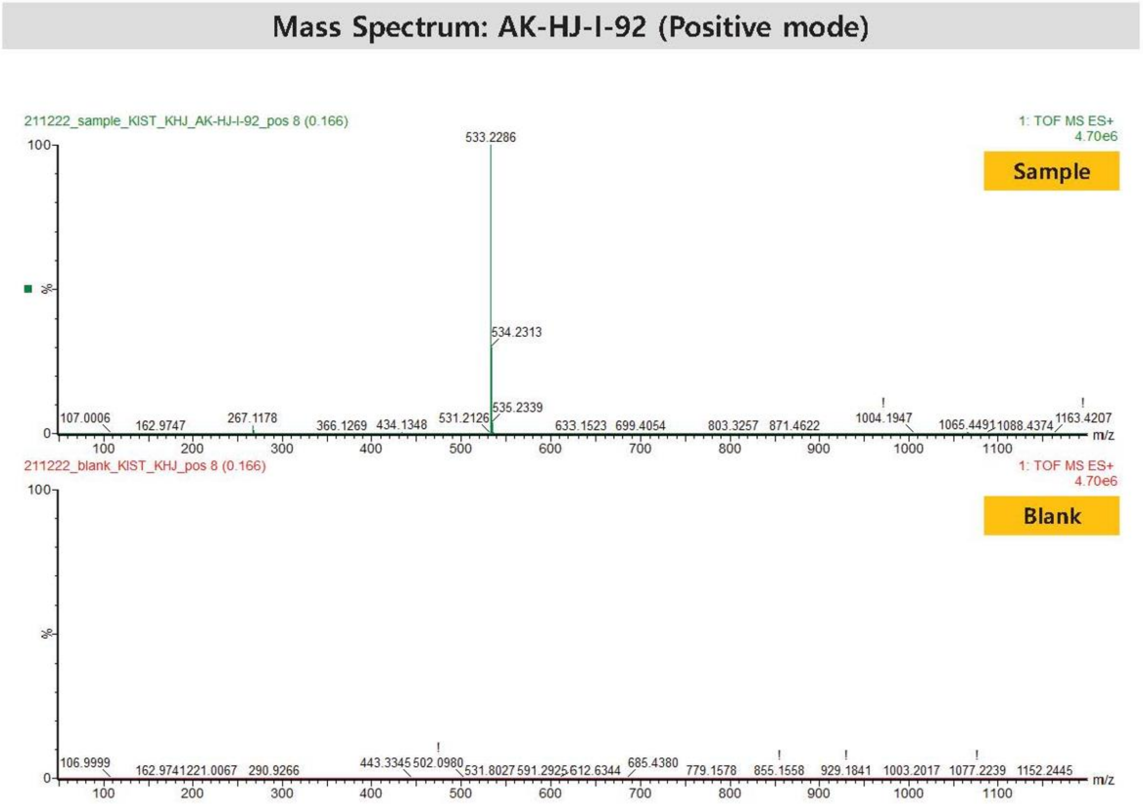

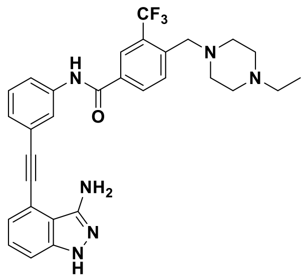

4b

$C_{30}H_{30}F_3N_6O$ $[M+H]^+$:

Calculated: 547.2433

Found: 547.2430

Mass Spectrum: AK-HJ-I-94 (Positive mode)

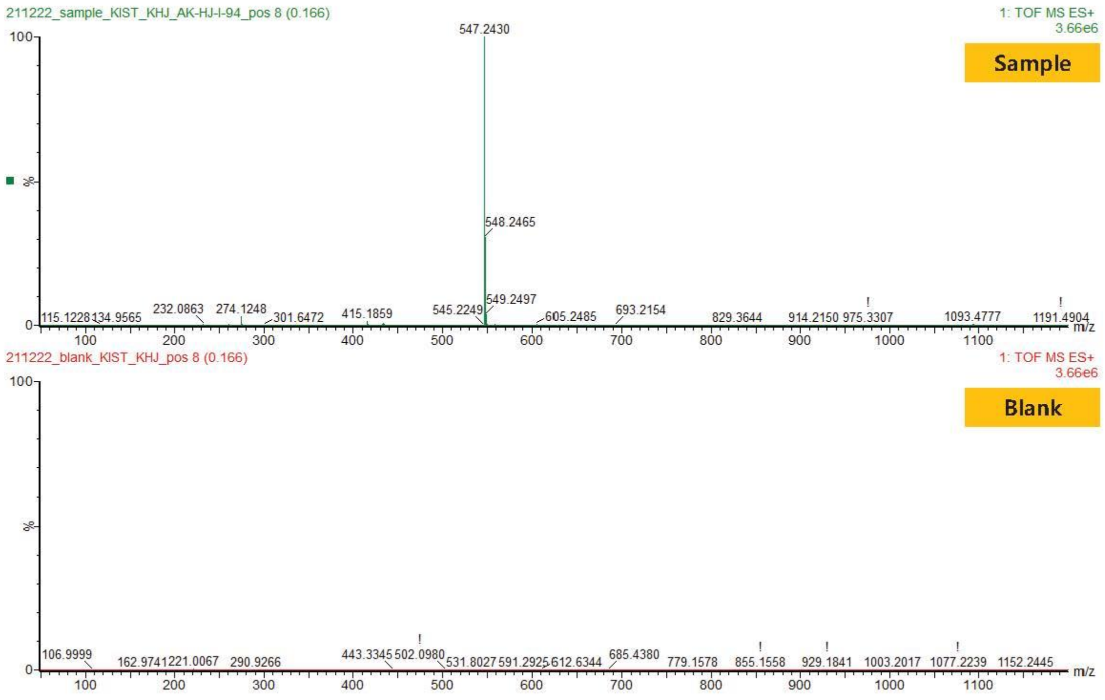

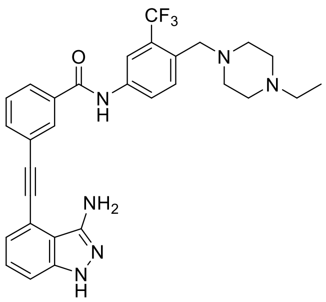

5

$C_{30}H_{30}F_3N_6O$ $[M+H]^+$:

Calculated: 547.2433

Found: 547.2438

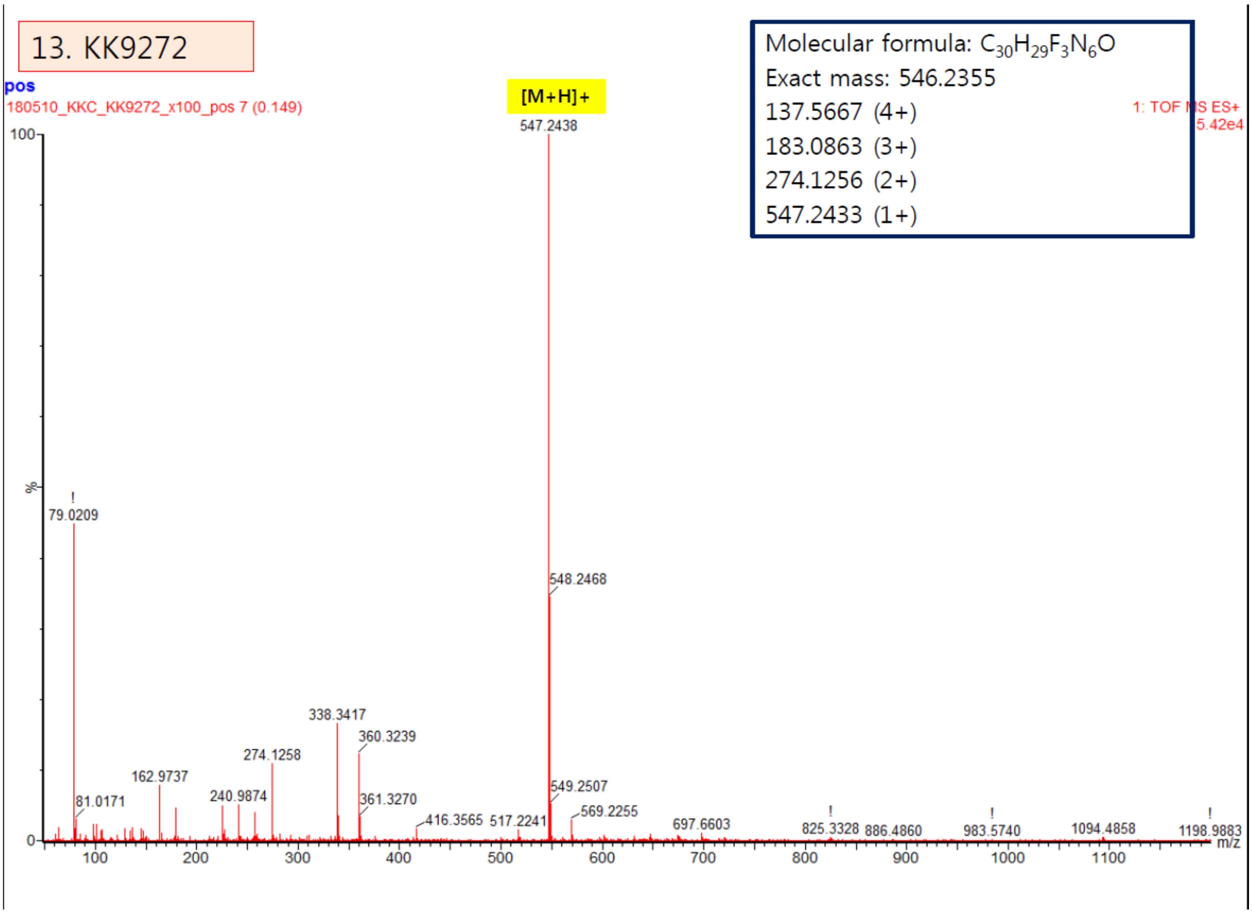

4) ADME-Tox prediction by pkCSM

Molecule Depiction

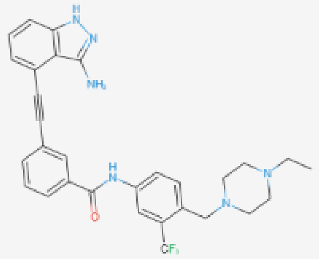

SMILES

Molecule properties:

Descriptor	Value
Molecular Weight	546.597
LogP	4.9535
#Rotatable Bonds	5
#Acceptors	5
#Donors	3
Surface Area	229.700

Property	Model Name	Predicted Value	Unit
Absorption	Water solubility	-3.019	Numeric (log mol/L)
Absorption	Caco2 permeability	0.832	Numeric (log Papp in 10^{-6} cm/s)
Absorption	Intestinal absorption (human)	84.945	Numeric (% Absorbed)
Absorption	Skin Permeability	-2.735	Numeric (log Kp)
Absorption	P-glycoprotein substrate	Yes	Categorical (Yes/No)
Absorption	P-glycoprotein I inhibitor	Yes	Categorical (Yes/No)
Absorption	P-glycoprotein II inhibitor	Yes	Categorical (Yes/No)
Distribution	VDss (human)	0.903	Numeric (log L/kg)
Distribution	Fraction unbound (human)	0.199	Numeric (Fu)
Distribution	BBB permeability	-1.015	Numeric (log BB)
Distribution	CNS permeability	-1.974	Numeric (log PS)
Metabolism	CYP2D6 substrate	Yes	Categorical (Yes/No)
Metabolism	CYP3A4 substrate	Yes	Categorical (Yes/No)
Metabolism	CYP1A2 inhibitior	No	Categorical (Yes/No)
Metabolism	CYP2C19 inhibitior	No	Categorical (Yes/No)
Metabolism	CYP2C9 inhibitior	No	Categorical (Yes/No)
Metabolism	CYP2D6 inhibitior	No	Categorical (Yes/No)
Metabolism	CYP3A4 inhibitior	No	Categorical (Yes/No)
Excretion	Total Clearance	0.536	Numeric (log ml/min/kg)
Excretion	Renal OCT2 substrate	No	Categorical (Yes/No)
Toxicity	AMES toxicity	No	Categorical (Yes/No)
Toxicity	Max. tolerated dose (human)	0.683	Numeric (log mg/kg/day)
Toxicity	hERG I inhibitor	No	Categorical (Yes/No)
Toxicity	hERG II inhibitor	Yes	Categorical (Yes/No)
Toxicity	Oral Rat Acute Toxicity (LD50)	2.311	Numeric (mol/kg)
Toxicity	Oral Rat Chronic Toxicity (LOAEL)	2.95	Numeric (log mg/kg_bw/day)
Toxicity	Hepatotoxicity	Yes	Categorical (Yes/No)
Toxicity	Skin Sensitisation	No	Categorical (Yes/No)
Toxicity	*T.Pyriformis* toxicity	0.285	Numeric (log ug/L)
Toxicity	Minnow toxicity	3.662	Numeric (log mM)

5) Molecular docking figures

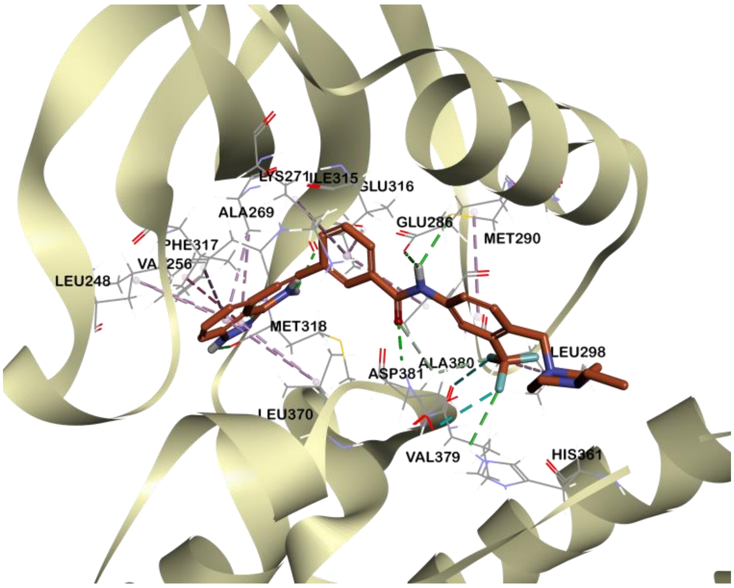

Figure S1. The binding mode of **5** in the BCR-ABL^{T315I} kinase (3OY3). The grey line shows interaction residue of BCR-ABL^{T315I} kinase. Various interactions are showed by different color.

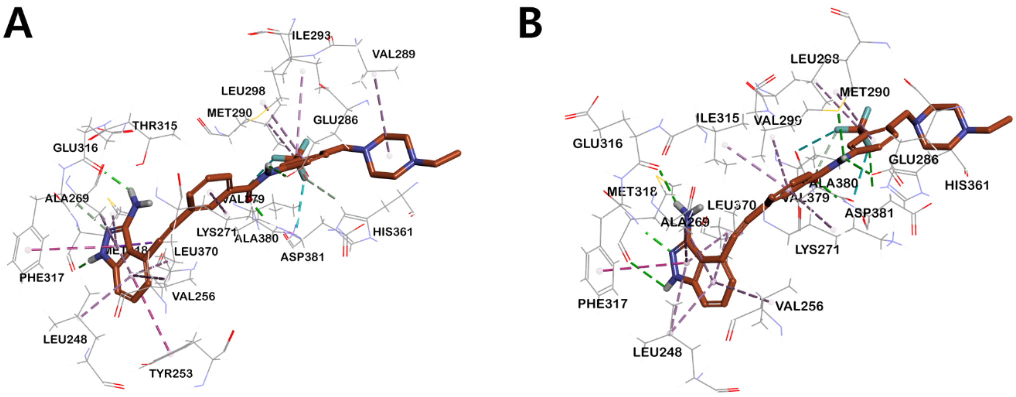

Figure S2. The binding mode of compound **5** in the (A) BCR-ABL^{WT}(3OXZ) and (B) BCR-ABL^{T315I} (3OY3) kinase. For clarity purpose, only residues having interactions were shown. Compound **5** are shown in stick model and surrounding residues were shown in line model. Various interactions are shown in dashes.

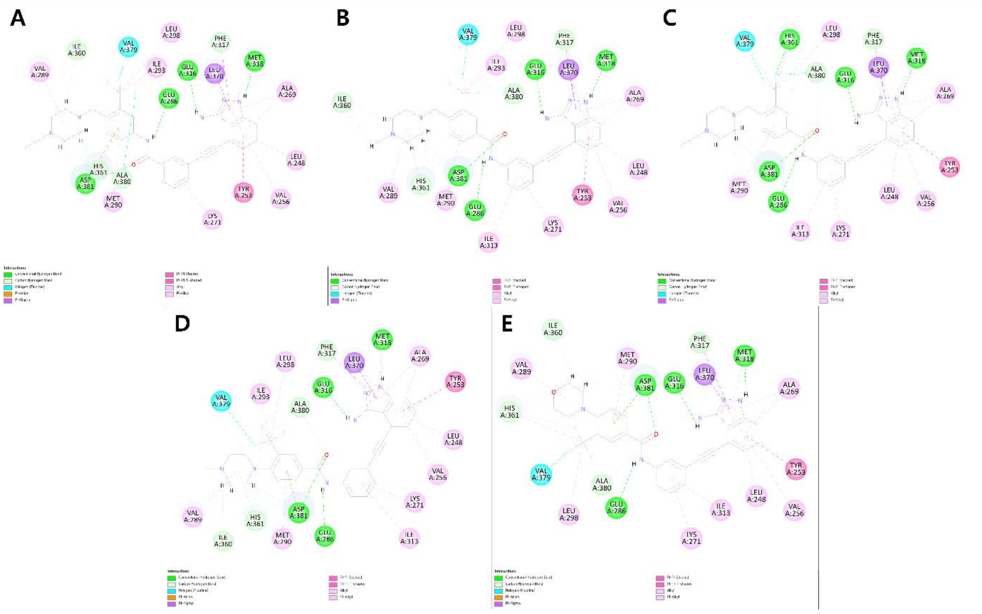

Figure S3. 2D-interaction docking model of (A) **5**, (B) **4a**, (C) **4b**, (D) **II** and (E) **I** inside BCR-ABL^{WT} (3OXZ). Various interactions are depicted by different color legends. Inhibitors are shown by line, interacting residues by colored sphere, and interactions by dash lines.

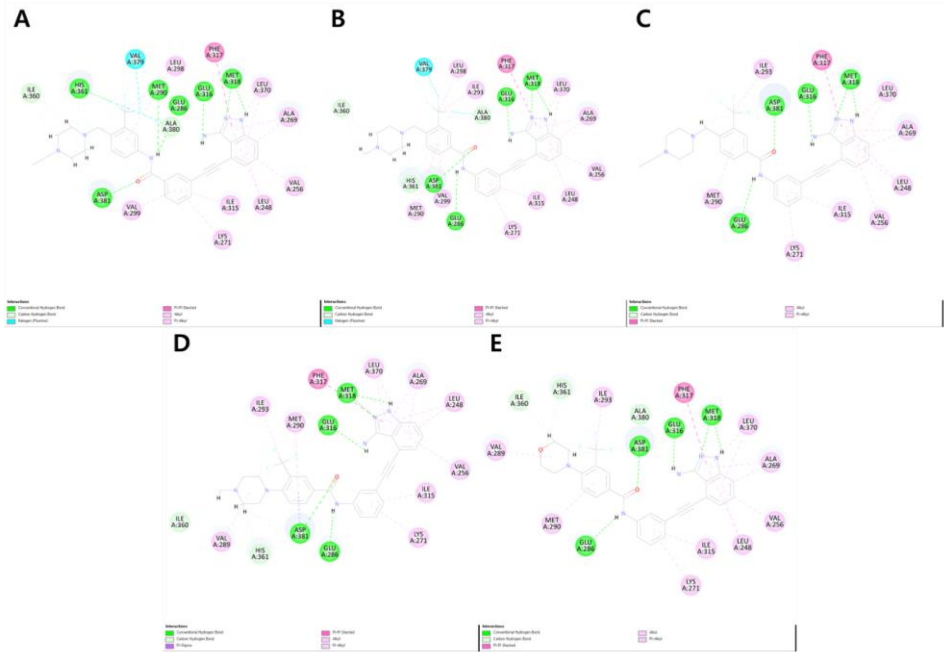

Figure S4. 2D-interaction docking model of (A) **23b**, (B) **22a**, (C) **22b**, (D) **9g** and (E) **9h** inside Abl^{T315I} (3OY3). Various interactions are depicted by different color legends. Inhibitors are shown by line, interacting residues by colored sphere, and interactions by dash lines.

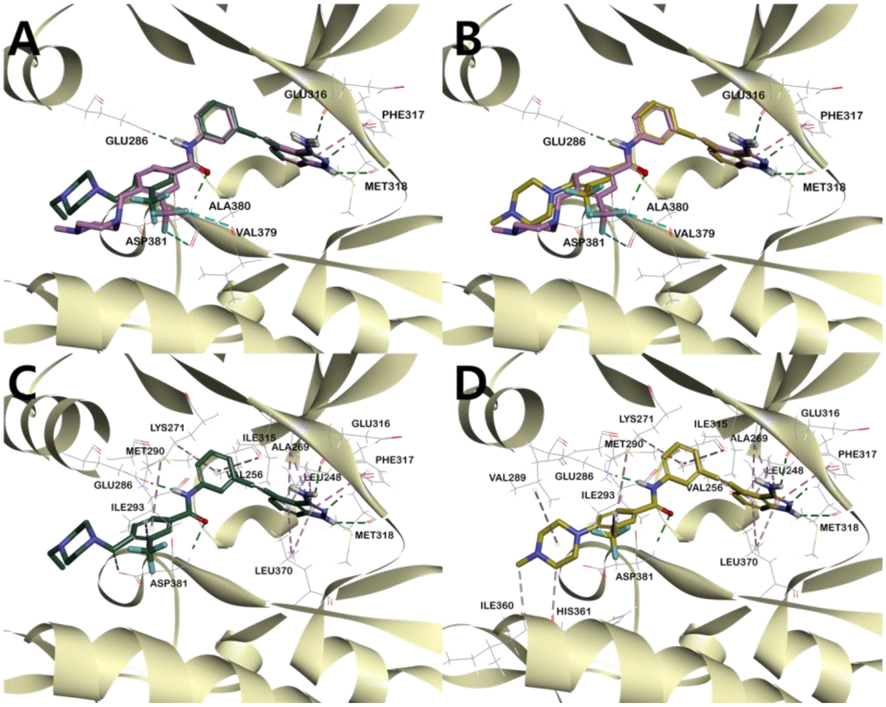

Figure S5. 3D structural overlay of compounds (A) **4a** (pink) and **4b** (green), (B) **4a** and **II** (yellow) in the BCR-ABL^{T315I} Kinase. Inhibitors are shown in stick model and surrounding key interaction residues were shown in line model. The key interaction mode between **4a** and BCR-ABL^{T315I} indicated by a dash line. The binding mode of compounds (C) **4b**, and (D) **II** in the BCR-ABL^{T315I} Kinase. Various interactions are shown in dashes.

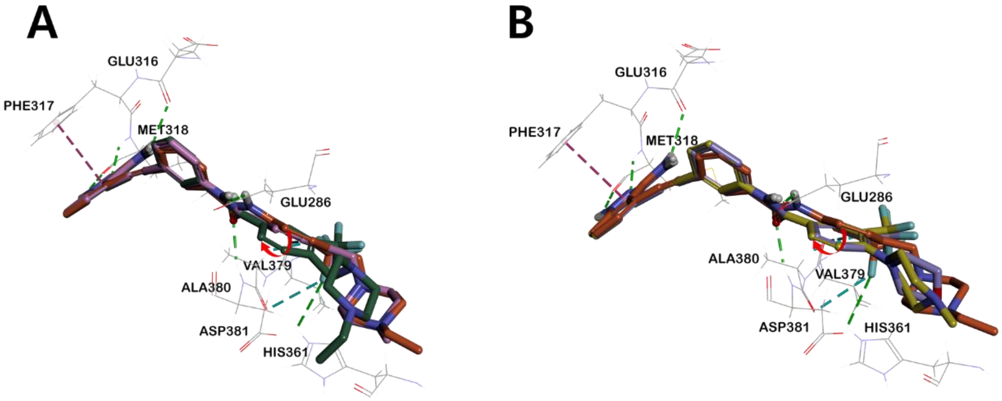

Figure S6. 3D structural overlay of compounds (A) **5** (orange), **4a** (pink) and **4b** (green), (B) **5** (orange), **II** (yellow) and **I** (lavender) in the BCR-ABL^{T315I} Kinase. Inhibitors are shown in stick model and surrounding key interaction residues were shown in line model. The key interaction mode between **4a** and BCR-ABL^{T315I} indicated by a dash line. The red curved arrow indicates the twisted angle of the trifluoromethylphenyl moiety.

6) References

1. Chen ML, Yuepeng; Wu, Dapeng; Sun, Yan; Cao, Jian. Process for preparation of 4-[(4-methylpiperazin-1-yl)methyl]-3-(trifluoromethyl)benzoic acid. 2018:Patent Number CN107663151.
2. El-Damasy AK, Cho NC, Nam G, Pae AN, Keum G. Discovery of a Nanomolar Multikinase Inhibitor (KST016366): A New Benzothiazole Derivative with Remarkable Broad-Spectrum Antiproliferative Activity. Chemmedchem. 2016;11(15):1587-95.
3. Dai YJ, Hartandi K, Ji ZQ, Ahmed AA, Albert DH, Bauch JL, et al. Discovery of N-(4-(3-amino-1H-indazol-4-yl)phenyl)-N'-(2-fluoro-5-methylphenyl)urea (ABT-869), a 3-aminoindazole-based orally active multitargeted receptor tyrosine kinase inhibitor. J Med Chem. 2007;50(7):1584-97.
4. Reaction Biology Corporation. Available from: http://www.reactionbiology.com/webapps/site/Kinase_Assay_Protocol.aspx [last accessed 14 November 2022].
5. DTP Human Tumor Cell Line Screen Process: Available from: https://dtp.cancer.gov/discovery_development/nci-60/methodology.htm [last accessed 14 November 2022].
6. Kuchukulla RR, Hwang I, Park SW, Moon S, Kim SH, Kim S, et al. Novel 2,6,9-Trisubstituted Purines as Potent CDK Inhibitors Alleviating Trastuzumab-Resistance of HER2-Positive Breast Cancers. Pharmaceuticals-Base. 2022;15(9).
7. Reaction Biology Corporation. Available from: https://www.reactionbiology.com/services/adme-safety/cytochrome-p450-assays [last accessed 14 November 2022].
8. Zhou TJ, Commodore L, Huang WS, Wang YH, Thomas M, Keats J, et al. Structural Mechanism of the Pan-BCR-ABL Inhibitor Ponatinib (AP24534): Lessons for Overcoming Kinase Inhibitor Resistance. Chem Biol Drug Des. 2011;77(1):1-11.
